# Proteomic Profiling of Cereal Aphid Saliva Reveals Both Ubiquitous and Adaptive Secreted Proteins

**DOI:** 10.1371/journal.pone.0057413

**Published:** 2013-02-27

**Authors:** Sohail A. K. Rao, James C. Carolan, Tom L. Wilkinson

**Affiliations:** School of Biology and Environmental Science, University College Dublin, Dublin, Ireland; Ghent University, Belgium

## Abstract

The secreted salivary proteins from two cereal aphid species, *Sitobion avenae* and *Metopolophium dirhodum*, were collected from artificial diets and analysed by tandem mass spectrometry. Protein identification was performed by searching MS data against the official protein set from the current pea aphid (*Acyrthosiphon pisum*) genome assembly and revealed 12 and 7 proteins in the saliva of *S. avenae* and *M. dirhodum,* respectively. When combined with a comparable dataset from *A. pisum*, only three individual proteins were common to all the aphid species; two paralogues of the GMC oxidoreductase family (glucose dehydrogenase; GLD) and ACYPI009881, an aphid specific protein previously identified as a putative component of the salivary sheath. Antibodies were designed from translated protein sequences obtained from partial cDNA sequences for ACYPI009881 and both saliva associated GLDs. The antibodies detected all parent proteins in secreted saliva from the three aphid species, but could only detect ACYPI009881, and not saliva associated GLDs, in protein extractions from the salivary glands. This result was confirmed by immunohistochemistry using whole and sectioned salivary glands, and in addition, localised ACYPI009881 to specific cell types within the principal salivary gland. The implications of these findings for the origin of salivary components and the putative role of the proteins identified are discussed in the context of our limited understanding of the functional relationship between aphid saliva and the plants they feed on. The mass spectrometry data have been deposited to the ProteomeXchange and can be accessed under the identifier PXD000113.

## Introduction

Insects that feed on living prey rely on bioactive compounds present in their saliva to negate the defences of their host. The saliva of animal and plant feeding insects alike comprises a diverse suite of proteins that suppress, circumvent, modulate and even induce host defence and immune responses. Whilst the saliva of blood feeding insects has been extensively studied, due in part to their role as vectors of mammalian disease e.g. [1]–[3], it is becoming increasingly clear that the salivary repertoires of plant feeding insects are equally complex. Aphids (Hemiptera: Aphidoidea) are phytophagous insects that feed on the phloem sap of plants. They are unusual herbivores because their feeding site is a single phloem cell in the sieve element buried deep within plant tissues, yet they represent one of the most important insect pests of temperate agriculture [4], [5]. The aphid mouthparts are modified into fine, needle-like stylets that can penetrate between plant cells and puncture individual cells, including the sieve element. Aphid feeding reduces plant fitness by removing photoassimilates, transmitting plant viruses and, in some cases, altering plant growth and development [6].

The intimate relationship between the aphid and plant is mediated by the secretion of copious amounts of saliva during all stages of feeding, including probing and ingestion [7]. The saliva is secreted as a liquid but during probing it hardens around the stylets to form a sheath that remains in the plant after the aphid has withdrawn the stylets. When the stylets are located within cells the saliva remains in liquid form (so-called ‘watery saliva’). The secretion of saliva is divided into four phases: (i) intercellular sheath secretion; (ii) intracellular salivation into cells along the stylet path; (iii) phloem salivation into sieve elements; and (iv) phloem feeding salivation i.e. feeding interspersed with sporadic periods of salivation [8], [9].

The composition of aphid saliva and the specific functions of salivary proteins have become clear only recently, driven primarily by the increase in available genomic resources for aphids, particularly the sequenced genome of the pea aphid *Acyrthosiphon pisum* (Harris, 1776) [10]. Salivary gland expressed sequence tag (EST) libraries for *A. pisum* and the green peach aphid *Myzus persicae* (Sulzer, 1776) have been exploited to provide candidate salivary proteins [11]–[13]. Subsequent characterisation of a number of these proteins has indicated their importance in facilitating the plant-aphid interaction [12], [14]. Mass spectrometry (MS)-based proteomics has also been used to identify proteins directly from saliva secreted into chemically-defined diets by *M. persicae* [15] and *A. pisum* [16]. These studies have identified metalloproteases, glucose oxidases, regucalcin, NADH dehydrogenase and several novel proteins lacking homologues outside aphids, including a putative constituent of the salivary sheath.

In this paper, we identify salivary proteins secreted into chemically-defined diets by *Sitobion avenae* (Fabricius, 1775) and *Metopolophium dihrodum* (Walker, 1849), two aphid species that are important agricultural pests of cereals in Europe [17], [18]. The results represent one of the first MS-based characterisations of the saliva of aphids that are restricted to feeding on plants from within the monocot family Poaceae, and are a test of the utility of using genomic information derived from *A. pisum* (see www.aphidbase.com) to identify proteins in other aphid species from peptide mass spectrometry. The identification and functional characterisation of aphid salivary proteins has ecological and applied implications since variation in salivary protein composition could be an important driver in plant acceptance, perhaps mediating aphid host plant range and the ability of specific genotypes to exploit different crop varieties. Salivary profiles from *S. avenae* and *M. dirhodum* are compared to those obtained for *A. pisum* [16] to identify common proteins secreted by the different aphids. cDNA sequencing of the common salivary genes informed the design of antigenic peptides to generate polyclonal antibodies for exploring tissue expression of the conserved proteins in the different aphid species.

## Materials and Methods

### Insect Material

Stock cultures of *S. avenae* (clone CGSA5) and *M. dirhodum* (clone CGMD3) were derived from field collections of aphids recovered from *Triticum aestivum* (cv. Granary) growing at UCD in June 2009. The aphids were maintained separately as asexual clonal lineages on *T. aestivum* (cv. Byron) at 20°C, 18L∶6D regime at a low density to minimise the production of alatae. An asexual clonal lineage of the pea aphid *Acyrthosiphon pisum* (clone LL01) was maintained under identical environmental conditions on *Vicia faba* (cv. The Sutton).

### Collection of Aphid Saliva

The secreted saliva from approximately 40,000 aphids was collected from chemically-defined diets as previously described [16] by pooling protein concentrates from multiple daily collections. In brief, 4000 aphids were distributed to 50 diet preparations and allowed to feed for 24 hours. A single diet preparation consisted of approximately 5 ml of chemically-defined diet (see [19] for full composition) sealed between two sheets of Parafilm membrane stretched over one end of a polyurethane ring (height 50 mm, internal diameter 90 mm). The aphids were removed and introduced to new diet preparations every day, with the density maintained by addition of new aphids from the culture when necessary. The suitability of the diet for the aphids was evidenced by copious honeydew excretion and the production of nymphs throughout the experimental period. Diet preparation and saliva collection were conducted under aseptic conditions using filtered (0.2 µm) cell biology grade endotoxin-free water (Sigma Aldrich, Ireland) with all plastics, including the supporting rings and Parafilm sheets surface sterilised and exposed to UV light for a minimum of one hour.

The diets from a single 24 hour collection period were pooled to give a volume of approximately 200 ml and concentrated at 4°C under nitrogen in a Vivacell 250 Gas Pressure Concentrator (Sartorious Mechatronics, UK) using a 5000 molecular weight cut-off polyethersulfone (PES) membrane. The concentrate (approximately 5 ml) was washed with 50 ml phosphate buffered saline and concentrated again to 5 mL as above, followed by further concentration using a Vivaspin 6 centrifuge concentrator (Sartorius Mechatronics, UK) with a 3000 molecular weight cut-off PES membrane. Non-protein contaminants were removed from the final concentrate using a 2-D clean-up kit (GE Healthcare, product no. 80-6484-51) and analytical replicates were prepared by combining the concentrated saliva from ten independent 24 hour collections. Proteins were separated by one dimensional SDS-PAGE (1-DE) and visualised with the PlusOne Silver Staining Kit (GE Healthcare, product no. 17-1150-01) omitting gluteraldehyde for compatibility with mass spectrometry. Gels were digitalised using a GS-800 calibrated densitometer coupled with the Discovery Series QuantOne software (v 4.4; Bio-Rad, Sweden).

### In Gel Sample Preparation for Mass Spectrometry

Visible protein bands were excised using sterile scalpel blades and prepared for mass spectrometry following a modified protocol [20]. Samples were digested overnight with 13 ng****µl^−1^ sequencing grade modified porcine trypsin (Promega, USA) in 50 mM ammonium bicarbonate, and peptides were extracted from the supernatant in 30% acetontirile/0.2% trifluoroacetic acid and then 60% acetonitrile/0.2% trifluoroacetic acid. Samples were dried under vacuum and resuspended in 0.1% formic acid.

### Mass Spectrometry and Database Searches

The 1-DE separated proteins were subjected to LC MS/MS on a Finnigan LTQ mass spectrometer (Thermo Fisher Scientific, UK) connected to a Surveyor chromatography system incorporating an auto-sampler. Tryptic peptides were purified using a Michrom Peptide C8 CapTrap trapping cartridge (Michrom Bio- Resources, CA), eluted off the trap and separated using a Biobasic C18 Picofrit column (New Objective, MA) at a flow rate of 100 nl min^−1^ and gradient of 3–40% acetonitrile over 40 min. All data were acquired with the mass spectrometer operating in automatic data-dependent switching mode. A zoom scan was performed on the five most intense ions to determine charge state prior to MS/MS analysis. Protein identification from the MS/MS data was performed using the TurboSEQUEST [21] algorithm in BioWorks v. 3.2 (Thermo Fisher Scientific) to correlate the data against ACYPIproteins v2.1, the official protein set of the pea aphid genome assembly (33291 predicted protein models; accessed November 2011) available at http://www.aphidbase.com/aphidbase/downloads. The genomic sequence and databases used for peptide/protein searches were produced and made available by the Human Genome Sequencing Centre at Baylor College of Medicine (www.hgsc.bcm.tmc.edu) and the International Aphid Genomics Consortium (IAGC; www.aphidbase.com). The following search parameters were used: precursor-ion mass tolerance of 1.5 Da, fragment ion toleranceof 1.0 Da with methionine oxidation and cysteine carboxyamidomethylation specified as differential modifications and a maximum of two missed cleavage sites allowed. Two filters were applied: XCorr vs. charge state (1, 2, 3 and 4 = 1.50, 2.00, 2.50 and 3.00 respectively) and peptide probability (p<0.001). Matches with multiple unique peptides and a cumulative XCorr >20 are reported. Numerous proteins were identified based on single peptide hits but these are not reported. The mass spectrometry proteomics data have been deposited to the ProteomeXchange Consortium (proteomecentral.proteomexchange.org) via the Proteomics Identifications Database (PRIDE) partner repository [22] with the dataset identifier PXD000113.

### Production of Saliva-associated GLD and ACYPI009881 Antibodies

To facilitate the design of antibodies for the saliva associated proteins ACYPI009881 and glucose dehydrogenase (GLD), partial mRNA sequences were obtained for *S. avenae, M. dirhodum* and *A. pisum.* RNA was extracted from 20 wingless adult aphids for *S. avenae* and *M. dirhodum* and from 2 wingless adult aphids for *A. pisum* using an RNaesy minikit (Qiagen, UK) following the manufacturer’s instructions for purification of total animal tissues. RNA was DNAse treated using DNase free (Ambion) following the manufacturer’s instructions. 1****µg of RNA was used for cDNA synthesis using the Superscript TM II Reverse Transcriptase kit (Invitrogen), 10 mM dNTPs (Invitrogen) and random hexamer primers (Invitrogen). Samples without reverse transcriptase were generated as a control for DNA contamination. Partial cDNA sequences for ACYPI009881 and both saliva associated GLDs were amplified by PCR using MegaMix blue (Microzone, UK) under the following PCR reaction conditions: denaturation at 94°C for 240 s followed by 32 cycles of 60 s at 94°C, annealing for 60 s, 60 s at 72°C and a final extension at 72°C for 420 s in a Peltier thermal cycler (PTC 200; MJ Research; see Table S1 for primer sequences and optimised annealing temperatures). Due to the identification of numerous GLD paralogues in the saliva of both cereal aphids and *A. pisum*, two saliva associated GLD genes, ACYPI005582 (GLD1) and ACYPI000113 (GLD2), were chosen for sequencing in an attempt to identify conserved regions that could be used to design antibodies to detect members of the GLD family rather than individual GLD proteins, which may vary qualitatively across different species. Triplicate PCR products were pooled and purified by agarose gel using the QIAquick gel extraction kit (Qiagen), following the manufacturer’s guidelines. DNA samples were Sanger sequenced using the commercial services of Macrogen Inc. (South Korea).

### Antibody Design

Antibodies were prepared commercially using peptides obtained from translated protein sequences for ACYPI009881 and GLD for *A. pisum, S. avenae* and *M. dirhodum*. Peptides were designed in consultation with, and antibodies were prepared using, the polyclonal antibody production services of Eurogentec (Belgium). The antigen chosen for GLD was designed from an almost fully conserved region between ACYPI005582 and ACYPI000113. Due to the length of ACYPI009881 and the presence of two imperfect repeats a double immunisation protocol (immunisation with 2 separate peptides) was adopted; peptides found in both repeated regions were chosen to improve the potential of antigen availability in subsequent immunodetection studies.

### Dissection of Salivary Glands and Protein Extraction

Salivary glands from adult aphids were dissected on sterile glass slides in ice-cold phosphate buffered saline (PBS, pH 7.0, Sigma-Aldrich, UK) using sterile fine needles (0.4 mm in diameter). To facilitate removal of the glands, the aphid was placed dorsal side down on the slide so that its mouthparts were facing upward. Both needles were inserted into the prothorax with a gap of about 5 mm separating them. The head was detached from the rest of the body by pulling one needle downward and the other needle upward. The separated head was held in place with a needle between the eyes and above the labrum and a second needle was inserted between the first pair of legs and moved upwards with a semi-circular action. At least one of the salivary glands was exposed at this point, usually attached to the other salivary gland and the brain. The salivary glands were gently excised from the surrounding head fragments and stored in batches of 20 glands at −80°C until further use.

Preliminary results indicated that approximately three hundred salivary glands were required for subsequent analysis following protein extraction. Salivary gland dissections were homogenised individually in PBST buffer (0.1% v/v Triton X–100 solution in PBS) and then pooled into a single sample. The sample was sonicated for 3×10 seconds and centrifuged at 9000 g for 10 minutes to pellet any debris. Aliquots of the supernatant were processed with a 2D clean up kit (GE, Healthcare) and the resulting protein pellet was re-suspended in 200 µl PBST.

### Western Blot Analysis

Western blotting was performed on secreted saliva and salivary gland proteins from *A. pisum, S. avenae* and *M. dirhodum* to verify the presence of ACYPI009881 and GLD in saliva and to localise the source of the synthesis of these common saliva associated proteins. For each blot, two saliva samples collected over 24 hours were pooled and resuspended in 30 µl PBST. 15 µl 3× loading buffer (New England Biolabs) and 10 µM DTT (New England Biolabs) were added to each sample prior to 1-DE (see above for additional conditions). Following electrophoresis, proteins were transferred from the gel to a nitrocellulose membrane using a wet transfer system (BioRad) at a voltage of 100V with 350 mA limit for 1 hour. Following transfer, the membranes were removed, washed in TBST buffer (0.05% Tween20 in TBS) and blocked with 5% Marvel dried milk in TBST. Membranes were incubated in primary antibody (1∶1000 dilution) overnight followed by extensive washing in TBST. The blots were transferred to an individual tray on a shaker and washed three times with TBST. The antigen-antibody complex was detected with horse radish peroxidase-conjugated goat anti-rabbit secondary antibody (Sigma-Aldrich, Ireland) at a 1∶5000 dilution, and subsequently using a metal enhanced 3,3-diaminobenzendine tetrachloride (DAB) substrate kit (Pierce, Ireland) following the manufacturer’s guidelines. To determine the specificity of the primary antibody for the protein band, antibodies at 1∶1000 dilution were blocked by preincubation with 5****µg/ml of the immunizing peptide. Two negative controls were performed: i) the blocked antibody was substituted for the primary antibody, and ii) the secondary antibody was substituted for the primary antibody.

### Immunohistochemistry Using Whole Salivary Glands

Individual salivary glands were dissected as previously and placed on 2% APTES (3-aminopropyltriethoxysilane in absolute alcohol) coated concave slides. The tissue was fixed in Bouin’s solution (BDH) for 10 minutes at room temperature and washed extensively with PBST. Two replicate groups of salivary glands were incubated with either primary antibody at 1∶100 dilution in PBST or blocked primary antibody (as negative control) overnight at 4°C. The secondary antibody was used in place of primary antibody on additional gland preparations to check for non-specific binding of the secondary antibody to the salivary gland tissue. Salivary glands that had been incubated overnight were washed repeatedly with ice cold PBST and salivary glands were blocked with 5% marvel milk (w/v) in PBST for 90 minutes at 4°C in the dark. Following repeated washes in ice-cold PBST the salivary glands were incubated with secondary antibody (FITC conjugated goat anti-rabbit, Sigma-Aldrich) at 1∶500 dilution in PBST for 5 hours at 4°C. After secondary antibody incubation, the salivary glands were washed extensively with ice-cold PBST. The glands were mounted in ice-cold PBST and observed under fluorescence using a Leica M 165 FC microscope with excitation and emission wavelength of 495 nm and 520 nm, respectively.

### Immunohistochemistry Using Sectioned Salivary Glands

Twenty salivary glands from each aphid species were dissected as previously and placed in a 0.5 ml microtube. The glands were fixed in Bouin’s solution and embedded following standard protocols [23] in paraffin wax (using a gelatine capsule to hold the sample) and serially sectioned using a hand microtome (cut4060, MicroTec) at a thickness of 5 µm. The sectioned glands were incubated either with primary antibody at 1∶100 dilution in PBST or with blocked antibody overnight at 4°C. Slides were washed with ice-cold PBST and tissue sections were blocked with 2% pre-immune serum in PBST for 60 minutes at 4°C. Sectioned glands were incubated with secondary antibody (FITC conjugated goat anti-rabbit) at 1∶500 dilution in PBST for 3 hours at 4°C. Subsequent immunodetection and visualisation steps were as described for whole salivary glands.

## Results

**Figure 1:**
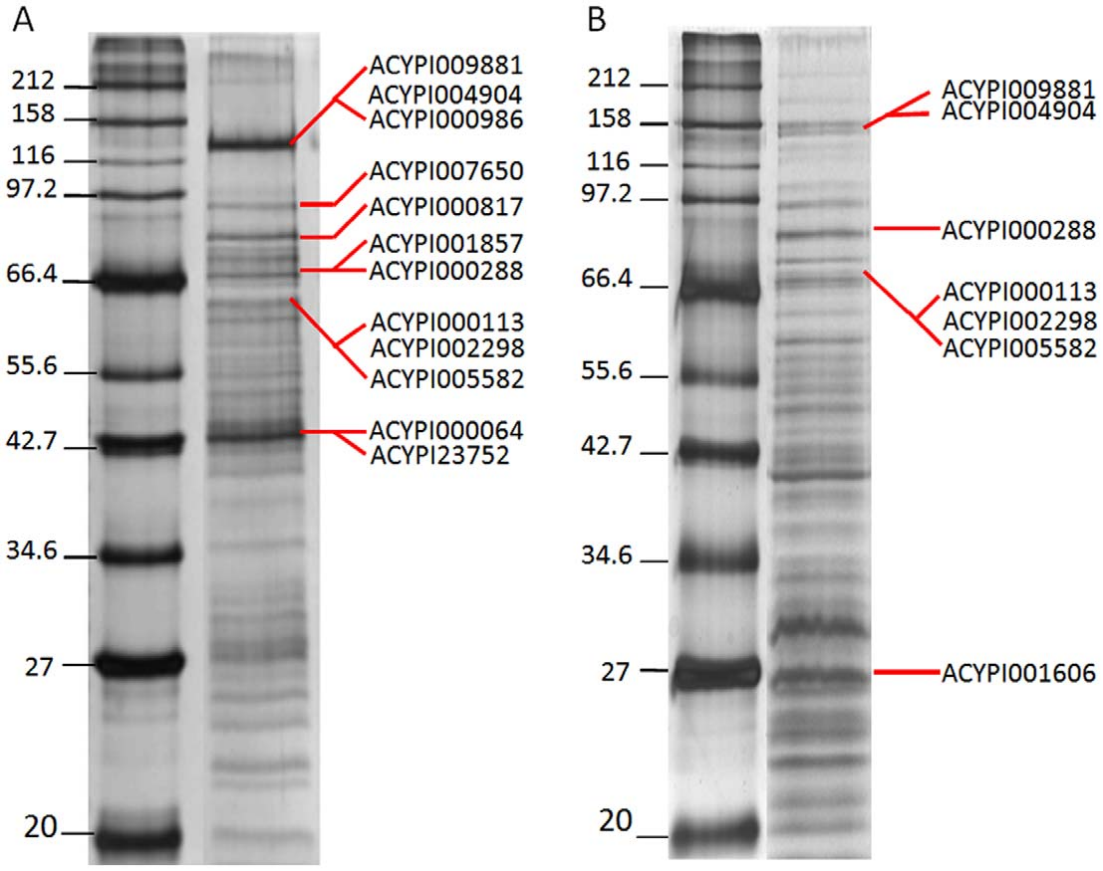
Fractionation of secreted salivary proteins by one-dimensional electrophoresis indicating bands from which proteins were identified by MS/MS and their subsequent protein hit/s (ACYPI numbers, see www.aphidbase.com). A) Salivary proteins secreted by *S. avenae*; 12 multiple peptide supported proteins were identified. B) Salivary proteins secreted by *M. dirhodum*; 7 multiple peptide supported proteins were identified.

The secreted saliva from the cereal aphids *S. avenae* and *M. dirhodum* was collected, concentrated and separated by one dimensional electrophoresis (1-DE) under semi-native conditions. Multiple, independent saliva collections consistently produced bands at approximately 148, 129, 80, 70, 66, 45 and 27 kDa in saliva from both aphid species (Figure 1). Additional bands that were detected in some samples sporadically were attributed to variable sample degradation and were not investigated further. Only bands that were observed consistently on the 1-DE gels were excised and analysed by LC-MS/MS. Proteins were identified in 6 bands from the saliva of *S. avenae* and in 4 bands from the saliva of *M. dirhodum*.

### LC-MS/MS and Protein Identification

Mass spectrometry data searched against the official protein set of *A. pisum* using the TurboSEQUEST algorithm (with a probability of less than 0.001 and over 70% amino acid similarity) resulted in the identification of 12 (46 peptides) and 7 (40 peptides) proteins in the saliva of *S. avenae* and *M. dirhodum,* respectively (Figure 1; Table 1 and Table 2). Of the 12 proteins identified in the saliva of *S. avenae*, four were glucose dehydrogenases (ACYPI000113, ACYPI005582, ACYPI000986 and ACYPI000288), peroxidase (ACYPI000817), trehalase (ACYPI002298), carbonic anhydrase (ACYPI23752), β-glucosidase (ACYPI007650), yellow e-3 like-protein (ACYPI001857), actin (ACYPI000064) and two non-annotatable proteins of unknown function (ACYPI009881 and ACYPI004904). The saliva of *M. dirhodum* comprised three glucose dehydrogenases (ACYPI000113, ACYPI005582 and ACYPI000288), trehalase (ACYPI002298) and three non annotatable proteins of unknown function (ACYPI009881, ACYPI004904 and ACYPI001606). Six of the seven proteins identified in the saliva of *M. dirhodum* were present in the salivary proteome of *S. avenae*.

**Table 1:** Peptide hits and identified proteins identified in the saliva of *Sitobion avenae.* The presence of a signal peptide and cleavage position was determined using SignalP 4.0.

Protein	ACYPI #	Peptides	Percentage coverage	Score XC	Peptide Probability	Expected MW (kDa)	Calculated MW (kDa)	Secretion Signal?	Cleavage Position
GMC Oxidoreductase	ACYPI000113	KFLTEQEDNLFKG KYDFDEWEKS RSVGYAMSDSTTRD KSAVGVEFVTKS KEMDVNVVADLPVGKN RKETFDLIPKG RASGNPDIEIMKI KNLATSIFHSVGTNKM	17.4%	100.21	5.70E-08	65	68	Yes	19–20
Hypothetical Protein	ACYPI009881	REYTVAVIGADDCWKE KKQVPLVYCYTRE RCGLNPNYLMKI KKAYEDIERS KNQIIASIIGYKD	5.2%	80.22	7.93E-10	129	129	Yes	16–17
Hypothetical Protein	ACYPI004904	KINAVVSMTVNYYQKK KTNVYASIRE KSVVHSNSDLAMRV RALNFNPDRS KSNMLSTSMDIPFKR	5.8%	60.23	1.47E-08	113	148	Yes	15–16
GMC Oxidoreductase	ACYPI000986	KGYVANTMSTTTTRL RSLNSVADWQFKI RGSEQLYNSLVKK KHLSEMEVPVVKD KLEDIDLDGCAKY	5.8%	50.20	1.28E-07	127	129	Yes	22–23
Peroxidase	ACYPI000817	RKQLDGSLLPIPRK RLITDVINENEYCPLRK RENVGVCFEAGDPRI RVGLDLSNNIRT	9.1%	50.23	1.77E-10	70	90	Yes	30–31
Carbonic anhydrase II	ACYPI23752	RYFGYWAFNRA RDMDNFIADLKY	8.3%	40.17	5.51E-06	29	50	Yes	17–18
Trehalase	ACYPI002298	KATNDFEYVKK KSAAETGWDFSSRW KAVASSVLGYLRD KLAQQVSFRL	8.1%	40.23	1.68E-08	68	68	Yes	20–21
GMC Oxidoreductase	ACYPI005582	KSPQLLMLSGIGPKE KINVFTNMFGYAKE KSVNPLDDPKI KLSNCADYIWDTRE	8.9%	30.18	2.93E-06	68	68	No	20–21
Beta-galactosidase precursor	ACYPI007650	RYVSGDLHYFRV RINYGDFIEDRK KGVAFLNDINLGRY	5.9%	30.18	4.20E-07	72	110	Yes	18–19
Yellow e-3 like Protein	ACYPI001857	LVVYDFK KSDEDFQFPSGMKI	4.6%	30.18	1.37E-06	49	72	No	32–33
Actin	ACYPI073036	KAGFAGDDAPRA KSYELPDGQVITIGNERF	8.0%	20.18	4.56E-11	41	45	No	23–24
GMC Oxidoreductase	ACYPI000288	KNVVHSVTTTRS KHYGPQIVPLKF	3.3%	20.18	3.59E-05	79	80	Yes	24–25
									

**Table 2:** Peptide matches and identified proteins in the saliva of *Metopolophium dirhodum.* The presence of a signal peptide and cleavage position was determined using SignalP 4.0.

Protein	ACYPI #	Peptides	Percentage coverage	Score XC	Peptide Probability	Expected MW (kDa)	Calculated MW (kDa)	Secretion Signal?	Cleavage Position
Hypothetical Protein	ACYPI009881	RDVNPDWSTYDKA RTGEVHMTRS KKQVPLVYCYTRE RCGLNPNYLMKI KYIIWVVTRD KKAYEDIERS KNQIIASIIGYKD	6.5%	120.22	6.33E-08	129	129	Yes	16–17
GMC Oxidoreductase	ACYPI000288	KNVVHSVTTTRS KCWSDLIDDKA KALSINTLEMCQKY KYGYNVEGLYVVPEFLRL KWSWEDVLKY KNHKGFVSSVAIVKN KMIGISLLRPKN KHYGPQIVPLKF KFEGGPEPDVDSDEYWKY	17.0%	100.22	5.89E-09	79	80	Yes	24–25
Hypothetical Protein	ACYPI004904	KLSEVTEVQQAQSILTARR RRCESSYINFMNDVTTIKT KTNVYASIRE KSTEVHYQRA KTVQLLQNSFKN KSNMLSTSMDIPFKR KTFLLSYLEKT RVDSINVMYSSVLIKS	10.1%	90.25	2.02E-10	113	148	Yes	15–16
GMC Oxidoreductase	ACYPI000113	KFLTEQEDNLFKG KYDFDEWEKS RKETFDLIPKG KGISTMGLTGLLSFVDSKR RHSDIILMIPISNIITKT RGIEFVVEMCKT	13.8%	70.26	4.53E-12	65	65	Yes	19–20
Trehalase	ACYPI002298	KQWALGLNQVWKT KATNDFEYVKK REDYESAEFLKT KNALLLSSWYSKM KAVASSVLGYLRD	10.5%	40.19	8.53E-08	68	68	Yes	20–21
Hypothetical protein	ACYPI001606	KFINQLGCKY KIIQGFIYSRA	9.2%	30.2	2.22E-05	25	25	Yes	24–25
GMC Oxidoreductase	ACYPI005582	KSPQLLMLSGIGPKE KINVFTNMFGYAKE KSMIDAGLVLEELKL	7.2%	10.22	1.43E-06	68	68	No	20–21

**Figure 2:**
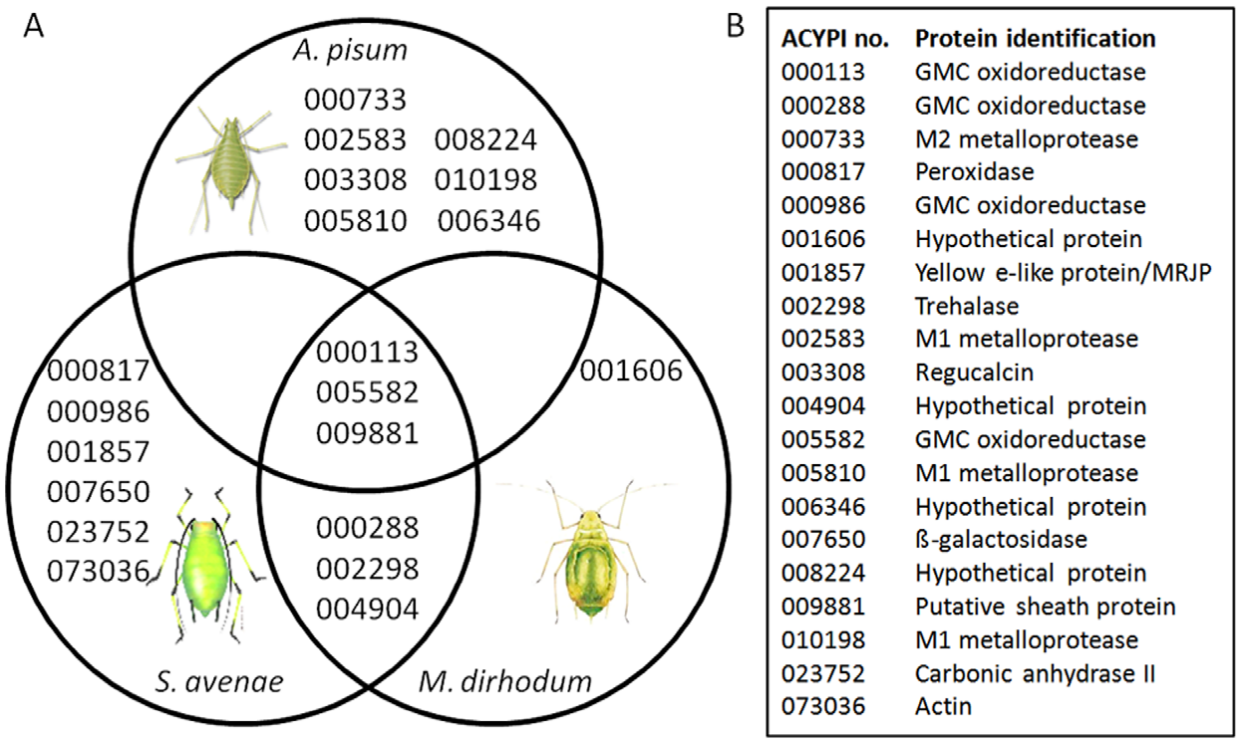
Distribution of proteins identified to date from the secreted saliva of *Acyrthosiphon pisum*, *Metopolophium dirhodum* and *Sitobion avenae.* Numbers in (A) are the ACYPI numbers referred to in (B) with their associated protein identification. Images not to scale.

SignalP analysis of the full length predicted protein sequences identified 9/12 and 6/7 sequences with N-terminal secretion signals from *S. avenae* and *M. dirhodum*, respectively (Table 1 and Table 2). An overview of the proteins identified in both species and a comparison to previously published MS-identified salivary proteins for *A. pisum* is given in Figure 2. Three proteins (ACYPI000113, ACYPI005582 and ACYPI009881) were detected in the secreted saliva of all three aphid species, and an additional 3 proteins (ACYPI000288, ACYPI002298 and ACYPI004904) were common to the secreted saliva of the two cereal aphids.

### RT-PCR and Antibody Design

RT-PCR was conducted on two GLDs (ACYPI000113 and ACYPI005582) and ACYPI009881, in order to determine sequences conserved between *S. avenae*, *M. dirhodum* and *A. pisum* for the design of antibodies. Two GLD genes were chosen due to the different levels of abundance between all three species. ACYPI005582 was the most abundant GLD in the saliva of *A. pisum* [16] whereas ACYPI000113 was the most abundant GLD in the saliva of *S. avenae* and *M. dirhodum.* 737 and 625 bps of the 3′- regions of the genes coding for the orthologues of ACYPI000113 and ACYPI005582, respectively, were amplified for both cereal aphids (GenBank Accession numbers JX417973–JX417976). Of the 5 antigenic peptides identified during the antibody design process (conducted by Eurogentec S.A. Belgium; data not shown), one peptide sequence (GGDPPESTENPLLW) was highly conserved across all three species for both genes and was subsequently chosen for antibody design (Figure S1). A 2420 bp region of the gene coding for ACYPI009881 was amplified for both cereal aphid species (Figure S2; GenBank Accession numbers JX417977–JX417978) and compared to the pea aphid orthologue. The two peptides chosen for antibody design (TTPCDDTDYNTEYEV and STYKKAYEDIERSGLC) are present within a c.450 amino acid repeated region within the pea aphid ACYPI009881 which results in 4 available epitopes for the anti-ACYPI009881 antibodies. Based on the gene sequences presented here, the ACYPI009881 orthologues of *S. avenae* and *M. dirhodum* also have this repeated region.

### Detection of ACYPI009881 and GLD in Secreted Saliva

**Figure 3:**
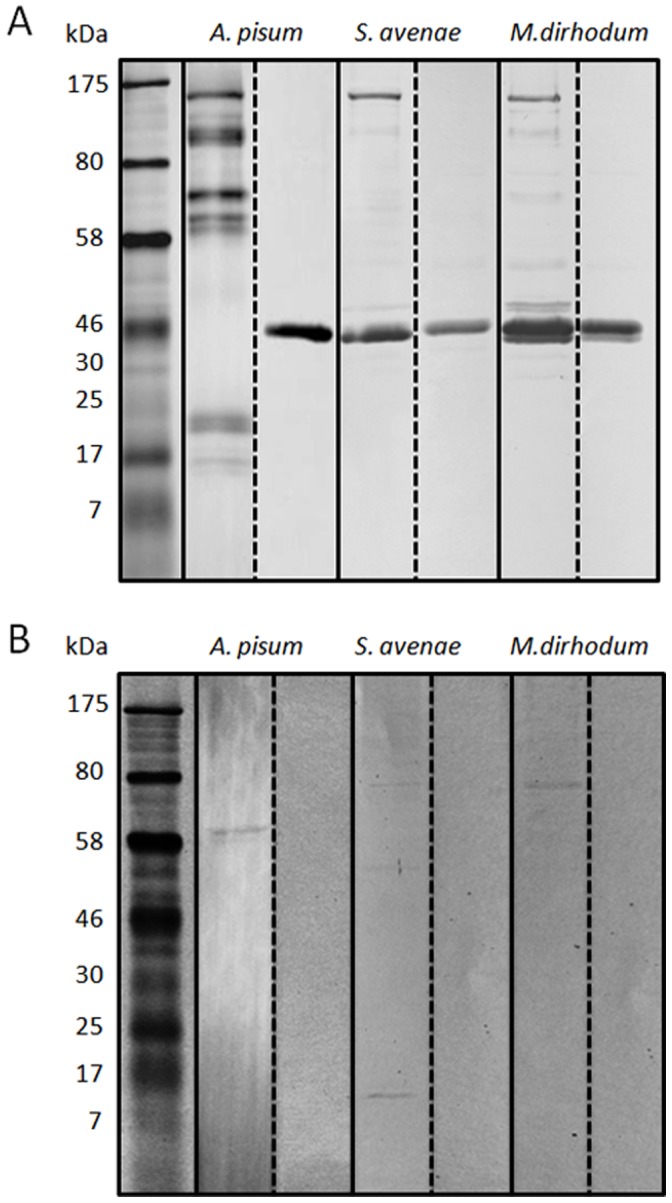
Immunoblotting of secreted salivary proteins from *A. pisum*, *S. avenae* and *M. dirhodum* using polyclonal antibodies raised against (A) ACYPI009881 and (B) saliva-associated GLD. Two parallel blots are shown for each aphid saliva/antibody combination; the left hand blot represents antibody-protein interactions whereas the right hand blot represents binding of the antibody after incubation with the immunizing peptide to demonstrate non-specific binding of the primary and/or secondary antibody. Blots in (B) were overexposed to visualise the faint bands representing antibody binding to saliva-associated GLD.

Western blot analysis was performed on secreted saliva collected from *A. pisum, S. avenae* and *M. dirhodum* using antibodies for ACYPI009881 (Figure 3A) and GLD (Figure 3B). Several bands were detected using anti-ACYPI009881 in the saliva of *A. pisum*, *S. avenae* and *M. dirhodum* including one at approximately 130 kDa which matches the region from which ACYPI009881 was excised and identified using mass spectrometry. Additional bands were observed on the blots for all species, indicating that the relatively large protein undergoes considerable degradation during the saliva collection and concentration process. A 45 kDa band of strong intensity was observed in the secreted saliva of *S. avenae* and *M. dirhodum.* However this protein was attributed to non-specific binding of either the primary or secondary antibodies as this band was also present in the negative controls using saliva and primary antibody that was blocked with the immunizing peptide.

The antibody for GLD detected three bands in the saliva of *S. avenae* at approximately 68 kDa, 50 kDa and 15 kDa (Figure 3B). No homologues for GLD that match the sizes of these additional bands were identified within the pea aphid official protein set indicating that they may simply represent degraded products of the higher molecular weight band. The band at 68 kDa matches the region excised from the gel from which both GLD proteins were identified using mass spectrometry. This 68 kDa band was also evident in the saliva of *M. dirhodum* again confirming the mass spectrometry results on the saliva for this species. Unexpectedly, western blots on the saliva of *A. pisum* comprised a single band at 58 kDa which is smaller than the proteins identified by mass spectrometry. The smaller size may be reflective of protein modification/processing or that an additional GLD is present in the saliva of *A. pisum* detectable by the antibody designed from ACYPI000113 and ACYPI005582. No bands were evident in the negative controls for all three species.

### Localisation of ACYPI009881 and GLD within Whole Salivary Glands

**Figure 4:**
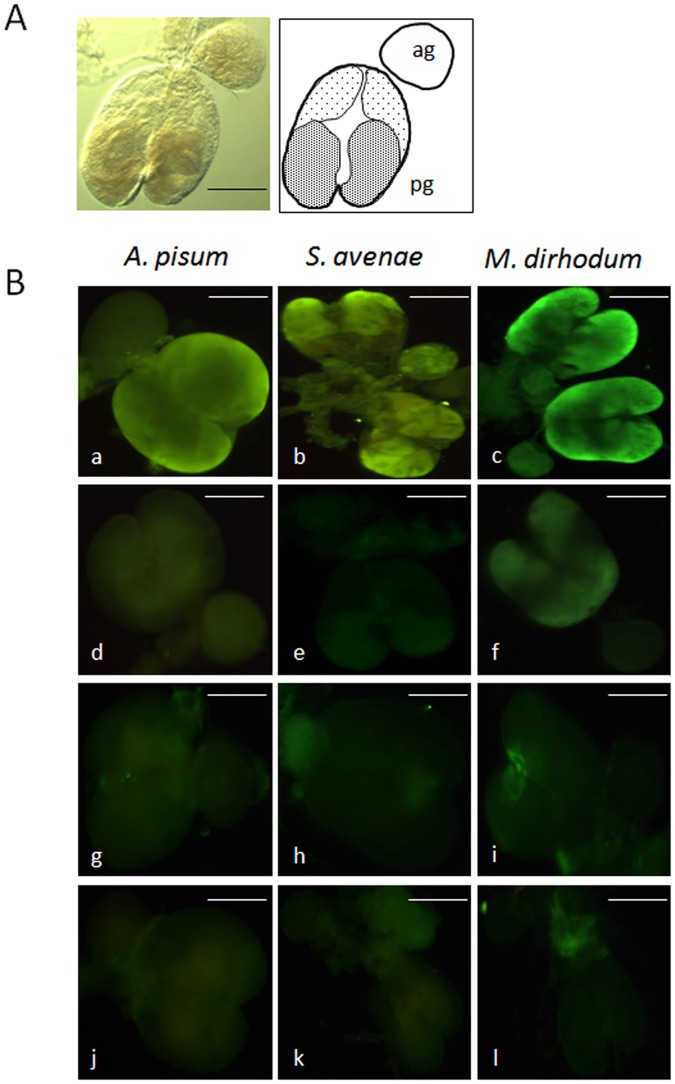
Aphid salivary gland morphology and immunohistochemistry (A) Freshly dissected salivary gland from *A. pisum* (left panel, x120, scale bar = 100 µm) and line representation (right panel) indicating principal gland (pg) and accessory gland (ag) and approximate delineation of hauptzpellen (dark stippling) and deckzellen (light stippling) regions of the principal gland. One half of the paired salivary gland is shown. See [25], [26] for further details of salivary gland morphology. (B) Immunolocalisation of secreted salivary proteins to whole salivary glands from *A. pisum*, *S. avenae* and *M. dirhodum*. Freshly dissected salivary glands were incubated with either anti-ACYPI009881 antibodies (a–c) or anti-ACYPI009881 antibodies blocked with immunizing peptides (d–f), and with anti-saliva-associated GLD antibodies (g–i) or anti-saliva-associated GLD antibodies blocked with immunizing peptides (j–l). Scale bar in all figures represents 100 µm.

The salivary glands of *S. avenae, M. dirhodum* and *A. pisum* had a similar morphology to previously described aphid salivary glands e.g. [24]–[26]. In brief, the glands are paired and consist of two principal glands and two accessory glands located between the head and pro-thorax. A large, bi-lobed principal gland joins with a smaller accessory gland to form one half of the gland and the two sides unite through the common salivary duct that leads to the mouthparts (one half of a freshly dissected salivary gland from *A. pisum* is shown in Figure 4A, together with a tissue map indicating areas of distinct morphology as described in [25]). Immunohistochemical analysis using anti-ACYPI009881 antibody localised the protein to the principal glands of all three species (Figure 4B a–c), confirming the salivary gland as the site of synthesis of ACYPI009881. The anti-ACYPI009881 antibody localised to the same specific areas in the salivary glands of all three species (corresponding to the hautzpellen region of [25]). Parts of the deckzellen region were also fluorescent, but at a lower intensity. When the salivary glands of all three species were incubated with primary antibody blocked with immunizing peptides, no fluorescent signal was evident from *A. pisum* and *S. avenae* (Figures 4B d,e) and a very weak signal was detectable in areas of the deckzellen region of the principal glands from *M. dirhodum* (Figure 4B f). No fluorescent signal was evident when the secondary antibody was used in place of primary antibody (Figure S3).

**Figure 5:**
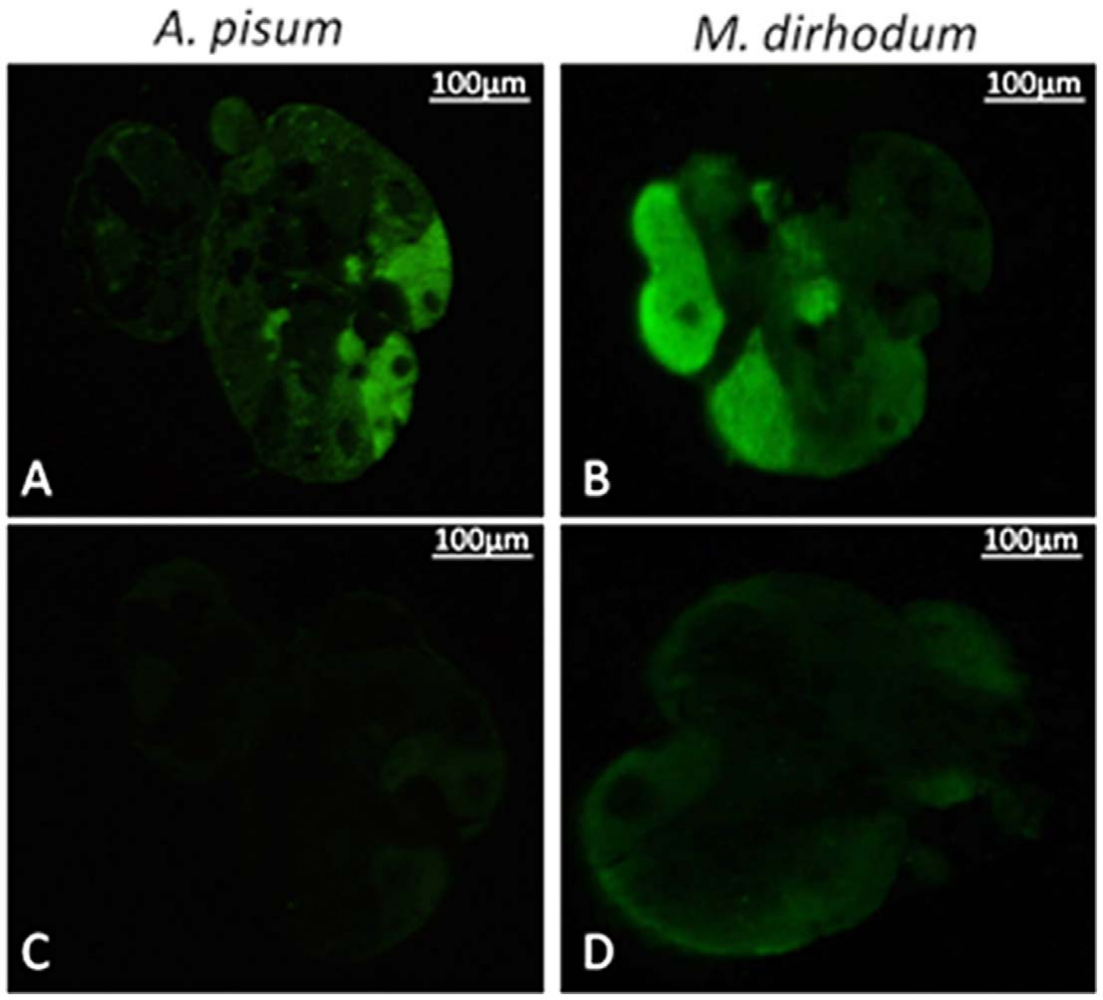
Immunolocalisation of ACYPI009881 within a single principal salivary gland from *A. pisum* and *M. dirhodum*. Tissue sections were incubated with either anti-ACYPI009881 antibody (A, B) or anti-ACYPI009881 antibody blocked with immunizing peptides (C, D). Scale bar in all figures represents 100 µm.

To determine more precisely the tissue location of ACYPI009881 within the hauptzellen region, immunolocalisation was performed on thin sections of salivary glands from *M. dirhodum* and *A. pisum*. ACYPI009881 was clearly localised to individual cells at the posterior end of the salivary gland (corresponding to cell types 5 and 6 of the classification scheme of [25]; Figure 5 a–b). No signal was detected in salivary gland sections of both species in which primary antibody was blocked with immunizing peptides and there was no fluorescence observed in the accessory salivary gland of either species (Figure 5 c–d).

*In situ* immunohistochemistry localisation of anti-GLD failed to detect significant levels of fluorescent signal in the salivary glands dissected from all three aphid species (Figure 4B g–i), although a diffuse signal was detected in glands from *M. dirhodum* (Figure 4B i) in a region comprising myoepithelial cells where the two lobes of the salivary gland join together [25]. No fluorescent signal was detected from the accessory salivary glands, and incubation with either anti-GLD blocked with antigenic peptides or with secondary antibody used as primary antibody resulted in no fluorescent signal (Figure 4B j–l and Figure S3).

## Discussion

The results presented here increase our understanding of the variability in bioactive salivary components secreted by aphids with broad scale differences in host plant preference. The salivary proteomes of two species of cereal aphid, *Sitobion avenae* and *Metopolophium dirhodum,* were characterised and compared to the salivary proteome of the model aphid species *Acyrthosiphon pisum*. Using both stringent search criteria and post probabilistic filter settings, 12 and 7 proteins were identified in the saliva of *S. avenae* and *M. dirhodum* respectively. Of the two cereal aphids, the saliva of *S. avenae* comprised a higher number of proteins, probably as a result of variation during saliva collection or technical variability among MS runs rather than any biological significance. However, of particular interest is the degree of similarity between the saliva of the two cereal aphids – six of the seven proteins identified in the saliva of *M. dirhodum* were present in the salivary proteome of *S. avenae* indicating that they may be conserved adaptations for the cereal/monocot feeding habit.

Three individual proteins, identified as two paralogues of the GMC oxidoreductase family and ACYPI009881, a putative principal component of the salivary sheath, were common to all the aphids examined in the present study (see Figure 2). Members of the glucose-methanol-choline oxidoreductase family (generally annotated as glucose dehydrogenase, GLD, or glucose oxidase, GOX) are the most frequently reported proteins identified by mass spectrometry from aphid saliva. These proteins have been detected in saliva from the green peach aphid *Myzus persicae* [15]; the pea aphid *A. pisum* [16], the Russian wheat aphid (RWA) *Diuraphis noxia* [27] and, here, the grain aphid *S. avenae* and the rose-grain aphid *M. dirhodum*. Both GLD and GOX were identified in the saliva of *M. persicae* [15; although the reported peptides were identical] and the presence of GOX was confirmed using substrate specific assays on salivary concentrates. Oxidoreductase activity has also been demonstrated using substrate-specific assays in the saliva of the spotted alfalfa aphid *Therioaphis maculate* and *A. pisum* [28]. Aphid salivary proteins have been proposed to have the capacity to degrade reactive oxygen species [29]–[30], which represent a ubiquitous plant defence response to invading pathogens, including insects, and the saliva associated oxidoreductases can be considered prime candidates for such a function. However eight GMC-oxidoreductase paralogues have been identified within the pea aphid genome [13], five of which are associated with aphid saliva or the salivary gland proteome. Considerable variation exists in protein length and amino acid sequence and many of the genes are under positive diversifying selection with respect to homologues in other insects, suggesting that additional functions for these saliva associated GMC-oxidoreductases should also be considered. GLD and GOX have roles in insect development and immunity [31], [32] and salivary GOX has been identified in the saliva of the tomato fruitworm *Helicoverpa zea* [33]–[36] and has been implicated in the modulation/suppression of plant hormone defence pathways. Salivary oxidoreductases have also been implicated in the detoxification of noxious phytochemicals, solidification of the aphid salivary sheath, control of cellular redox state and scavenging of reactive oxygen species (ROS) [16], [19], [37]. Further effort is required to characterise this seemingly ubiquitous, abundant and potentially multifunctional group of salivary proteins.

The other protein identified as common to the saliva of the three aphids studied here was the aphid specific protein ACYPI009881. This protein of approximately 130 kDa comprises two near perfect repeats of 439 and 432 residues respectively, and is relatively rich in cysteine (4%), serine (11%) and threonine (10%) and is a proposed component of the salivary sheath that gels once secreted into plant extracellular space [16]. cDNA sequencing confirmed that the 2-repeat form of this gene are present in *M. dirhodum* and *S. avenae* as a section of the second repeated region was amplified. The salivary sheath is believed to contribute to the molecular concealment of feeding aphids from plant defences, including preventing leakage of sieve element contents into the apoplast, a known trigger of plant defences [16], [38]. Recent RNAi targeting of ACYPI009881 in *A. pisum* resulted in the failure of the salivary sheath to solidify correctly (T. Will, University of Giessen, personal communication). However, the presence of ACYPI009881 does not appear to be ubiquitous – mass spectrometry failed to detect the protein from the secreted saliva of the black bean aphid *Aphis fabae* or the bird-cherry oat aphid *Rhopalosiphon padi* [39], [40] suggesting that the protein may be restricted to the tribe Macrosiphini within the Aphidinae. In addition, ACYPI009881 was not detected in the saliva of the Russian wheat aphid *Diuraphis noxia* [27], a basal member of the Macrosiphini, but this may be due to insufficient protein for mass spectrometry [41] or the search strategy adopted [27; in which only a single protein of unknown function was reported).

The conserved nature of members of the GMC-oxidoreductase family and ACYPI009881 across most species studied to date provided the rationale for the design and production of antibodies to conduct additional validation and characterisation. Although the GLD antibodies were designed from an antigenic peptide that should have identified two of the four identified GLDs, neither could be localised definitively to the salivary gland of the three species studied. However GLD was detected on western blots of the saliva of all three species matching to the molecular weight region from which the excised proteins were identified using mass spectrometry. The characterisation of the pea aphid salivary gland proteome/transcriptome [13] also indicated low levels of these particular proteins although other members of the family were more abundantly represented. The simplest explanation is that a non-salivary gland origin of at least some of the secreted salivary proteins should be considered. The accessory gland filters fluid from haemolymph into the salivary gland [29], [42] and proteins from the haemolymph have previously been detected in the salivary gland and saliva in aphids [29]. Insect haemolymph is a complex mixture of proteins that originate from multiple sources including the fat bodies, haemocytes, reproductive organs and the gut. Considering that the closely related gall forming aphids of the Phylloxeridae are thought to utilise their salivary glands in an excretory capacity [43] it is not unreasonable to assume that a considerable proportion of the salivary proteome may not originate in the salivary gland. Further evidence for a non-salivary gland origin for secreted salivary proteins exists for the putative calcium binding protein regucalcin. This protein was identified in the saliva of the pea aphid [16] but was absent from the salivary gland proteome and/or transcriptome [13]. Regucalcin is homologous to anterior fat body protein found exclusively in the fat bodies of the flesh fly *Sarcophaga peregrine* [44]. Interestingly, a weak fluroescent signal was detected from the posterior region of the salivary glands of *M. dirhodum* when probed with anti-GLD antibodies, suggesting this area of myoepithelial tissue may be an important conduit for the transit of haemolymph components into the salivary gland. Taken together these results are particularly relevant to the ongoing search for putative effector proteins and their characterisation *in planta* since additional sources of these proteins should be considered.

In contrast to GLD, immunohistochemistry with antibodies raised against ACYPI009881 demonstrated that this protein was extremely abundant in the principal salivary glands of *A. pisum, S. avenae* and *M. dirhodum*. In addition, western blots confirmed the presence of the protein in the secreted saliva from all three species. ACYPI009881 was primarily localised to the hauptzellen region of the salivary glands although some fluorescence was observed in the deckzellen region. Immunolocalisation of anti-ACYPI009881 on sectioned salivary glands for *A. pisum* and *M. dirhodum* indicated that ACYPI009881 expression was localised to individual cells corresponded to cell types 5 and 6 within the hauptzellen region of the principal gland [25]. The cells exhibiting expression of ACYPI009881 differ from those that express another saliva associated protein C002 [14] adding further support to the view that specific regions or individual cells within the gland are responsible for the expression of different salivary proteins.

Trehalase was identified in the saliva of both *S. avenae* and *M. dirhodum* and has also been recently detected in the secreted saliva of *D. noxia* [27] which may indicate a common function within aphids restricted to feeding on cereals and other monocots. Although the main transport sugar in plants is sucrose, the less abundant disaccharide trehalose is also transported via phloem and is thought to function primarily as a signalling molecule, see [45]. Trehalase is responsible for the hydrolysis of trehalose to glucose and is also associated with environmental stress responses [46], [47]. The secretion of trehalase into the plant host may provide a mechanism for the aphid to disrupt a potentially threatening signal transduction system. Considerable variation exists among virulent and avirulent biotypes of *D. noxia* at the proteomic and mRNA transcript/sequence levels [27], [48] suggesting that further studies into the putative role of trehalase as a secreted aphid effector are warranted.

Peroxidases have long been associated with aphid saliva, see [30] and have been identified in saliva of numerous aphids using substrate specific assays [28], [49], [50] including *S. avenae* [51]. Potential functions for an *in planta* secreted peroxidase include the detoxification of phytochemicals and the control of ROS such as hydrogen peroxide, an antioxidant produced in response to biotic attack and cellular damage [52]. Hydrogen peroxide is a ubiquitous signalling molecule of phytohormone defence cascades in particular, see [53], [54]. The identification of peroxidase from the saliva of *S. avenae,* in addition to the proteins discussed previously may indicate the complexity of the aphid-plant interaction and the specific interacting pathways that have evolved to ensure uninterrupted feeding. As an example, if the salivary glucose oxidases encounter glucose [perhaps hydrolysed from trehalose by salivary trehalase] gluconic acid and hydrogen peroxide would be formed, the latter being potentially oxidised by peroxidase.

Four additional annotatable proteins were identified in the saliva of *S. avenae* including a yellow e-3 like protein, a carbonic anhydrase, β-galactosidase and actin. Some of these proteins were also identified in the saliva of *M. dirhodum* but they were not considered further since their identification was based on single peptides. Previous proteomic studies on aphid saliva have reported proteins based on single peptide hits [15], [27], [41] but many of these may represent false positives. Considering that significant experimental effort may be exhausted to further characterise proteins identified in the saliva e.g. [12], [55] it is essential that only proteins identified with a high degree of certainty are reported. β-galactosidase is a glycosyl hydrolase that hydrolyses terminal, non-reducing β-D-galactosyl residues from carbohydrates, glycoproteins and galactolipids. Suggested functions include the degradation of cell walls to aid intercellular stylet penetration similar to saliva associated pectinases or cellulases [30], [50]. Although most commonly associated with social insects yellow-e3-like proteins have been identified across bacteria, insects and fungi. This multifunctional gene family has been associated with pigmentation [56], [57], behaviour [57], [58], sex-specific reproductive maturation [59] and caste determination in honeybees [60]. A recent phylogenetic analysis [61] identified 14 paralogues of yellow-e3-proteins in the pea aphid and to our knowledge this is the first association of these proteins with insect saliva. The involvement of yellow-e3 protein in melanin formation leads to the speculation that this protein could be involved in regulating phenoloxidase defence mechanisms. Carbonic anhydrases are zinc-containing enzymes that reversibly catalyse the hydration/dehydration of carbon dioxide and bicarbonate in most complex organisms. Its activity contributes to the transfer and accumulation of H^+^ or HCO_3_^–^ in bacteria, plants, vertebrates and invertebrates [62] and results in a mechanism for control of pH homeostasis. Changes in cellular pH is thought to influence and direct the aphid towards the phloem since aphids prefer neutral or slightly alkaline phloem conditions [63]. Secreted carbonic anhydrases could potentially regulate the pH of the plant tissue surrounding the mouthparts, including the phloem. Actin is a highly conserved structural protein, present in both cytoskeleton and muscle and contributes to a variety of cellular events such as organelle transportation, exo- and endocytosis [64] and the presence of actin in the saliva may contribute to the structural integrity of the salivary sheath.

Two proteins of unknown function were also identified; ACYPI004904 which was present in the saliva of both species and ACYPI001606 which was present in the saliva of *M. dirhodum*. ACYPI004904 is a relatively large protein (1114 residues), has an n-terminal secretion signal and like the putative sheath protein ACYPI009881 it is relatively rich in serine (13.5%) and threonine (14%) residues indicating that this protein is potentially susceptible to multiple phosphorylation events once secreted. The potential for *in planta* post-translational modification of secreted aphid proteins opens up a new dimension in the aphid-plant interaction.

Aphid saliva comprises a diverse protein repertoire that is slowly revealing the intricate biomolecular interactions that underpin the successful colonisation of the host plant. The secreted salivary proteins detoxify phytochemicals, modulate and evade host defences, regulate the cellular environment and contribute to the biophysical structures required for phloem feeding. One of the benefits of mass spectrometry based proteomics as adopted here is that no *a priori* knowledge is required to detect proteins in a particular sample. However our increasing appreciation of the diversity of these secreted salivary proteins from different aphid species now requires additional approaches aimed at the functional characterisation of individual salivary components to fully understand the intricacies and complexities of this extraordinary interaction.

## Supporting Information

Figure S1 **Predicted amino acid alignment for the two saliva associated GLD paralogues for** ***A. pisum*** **(ApGLD-1 and ApGLD-2),** ***S. avenae*** **(SaGLD-1 and SaGLD-2) and** ***M. dirhodum*** **(MdGLD-1 and MdGLD-2).** The box indicates the antigenic peptide sequence that was chosen for antibody design.
(DOCX)

Figure S2 **Predicted amino acid alignment for ACYPI009881 for** ***A. pisum*** **,** ***S. avenae*** **and** ***M. dirhodum*** **.** The boxes indicating the antigenic peptide sequences chosen for antibody design are highlighted.
(DOCX)

Figure S3 **Localisation of SHP (A–C) and GLD (D–F) using secondary antibody as primary antibody on glands; (scale 100 µm for all pictures at 120×).**
(DOCX)

Table S1 **Primer sequences and optimised annealing temperatures for** ***M. dirhodum*** **and** ***S. avenae*** **saliva associated GLDs and putative sheath protein.**
(DOCX)
