# Correction: Proteomic profiling of cereal aphid saliva reveals both ubiquitous and adaptive secreted proteins

**DOI:** 10.1371/journal.pone.0304429

**Published:** 2024-05-23

**Authors:** Sohail A. K. Rao, James C. Carolan, Tom L. Wilkinson

In Fig 3B of this article [1], lanes 3, 5, and 7 (the right side lanes for *A*. *pisum*, *S*. *avenae*, and *M*. *dirhodum*, respectively) were incorrect and should not have been used in this figure.

**Fig 3:**
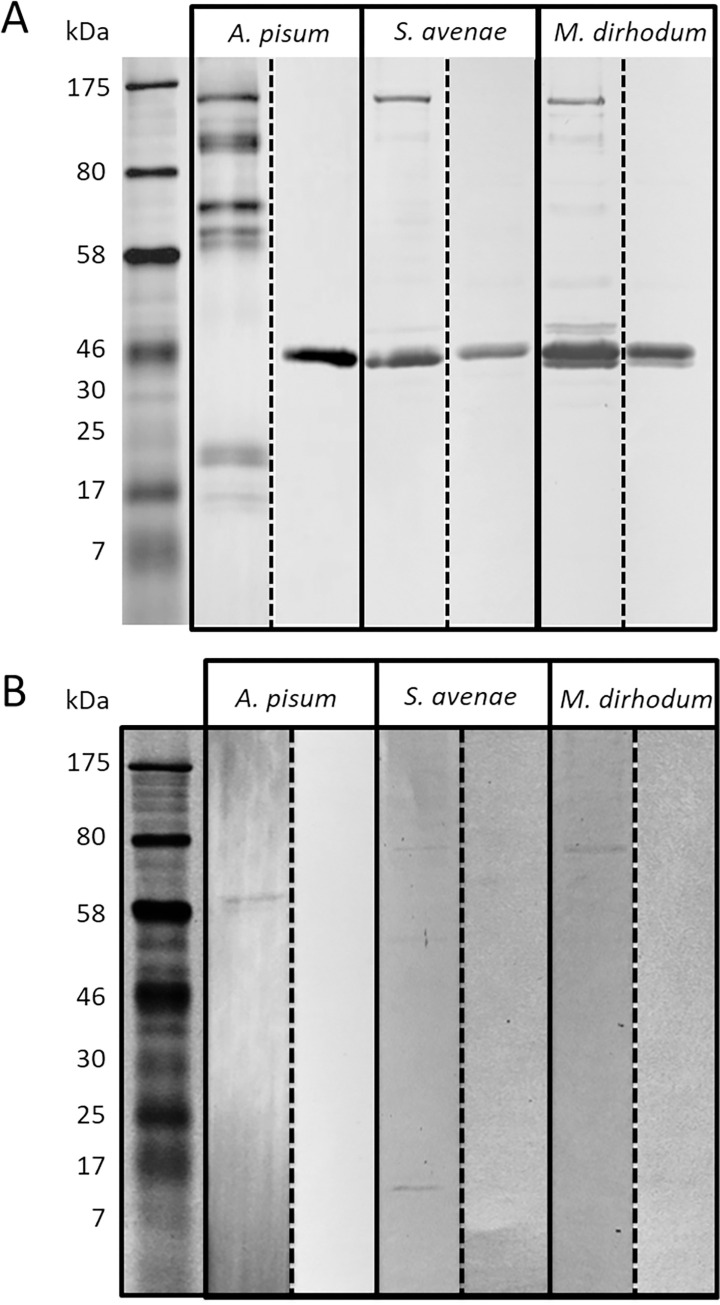
**Immunoblotting of secreted salivary proteins from *A*. *pisum*, *S*. *avenae* and *M*. *dirhodum* using polyclonal antibodies raised against (A) ACYPI009881 and (B) saliva-associated GLD.** Two parallel blots are shown for each aphid saliva/antibody combination; the left hand blot represents antibody-protein interactions whereas the right hand blot represents binding of the antibody after incubation with the immunizing peptide to demonstrate non-specific binding of the primary and/or secondary antibody. (The right and left lanes for each species were obtained using different blots.) The original blots in (B), except for *A*. *pisum* saliva, were overexposed to visualise the faint bands representing antibody binding to saliva-associated GLD.

In S3 Fig, the A and D panels were duplicates; the A panel is correct in this instance.

With this Correction, we provide updated versions of Fig 3 and S3 Fig in which the erroneous panels have been replaced with the correct data from the original experiments. The underlying data supporting the corrected figures are provided in S1 File. Whilst original uncropped copies of the raw blots derived from experimental equipment in Fig 3 were no longer available, the underlying images provided to support the correction were obtained from contemporary sources. Underlying images for S3 Fig are provided in S2 File.

The original SDS-PAGE images used in Fig 1 are no longer available. Sequences for ACYPI000113 and ACYPI005582 are available on NCBI via GenBank Accession numbers JX417977 and JX417978, respectively.

The mass spectrometry proteomics data are available on the ProteomeXchange Consortium (proteomecentral.proteomexchange.org) via dataset identifier PXD000113.

Underlying data for Figs 4 and 5 can be made available upon request.

The authors apologize for the errors in the published article.

## Supporting information

S3 Fig Localisation of SHP (A–C) and GLD (D–F) using secondary antibody as primary antibody on glands; (scale 100 µm for all pictures at 120×).
(TIF)

S1 File Underlying Fig 3 blot data. (PPTX)

S2 File Underlying images for S3 Fig. (ZIP)
