# Supplementary figures and images for: Proteomic Profiling of Cereal Aphid Saliva Reveals Both Ubiquitous and Adaptive Secreted Proteins
Source: PLoS One. 2013 Feb 27;8(2):e57413. doi: 10.1371/journal.pone.0057413 (PMC3584018; doi:10.1371/journal.pone.0057413)

**Figure S1**


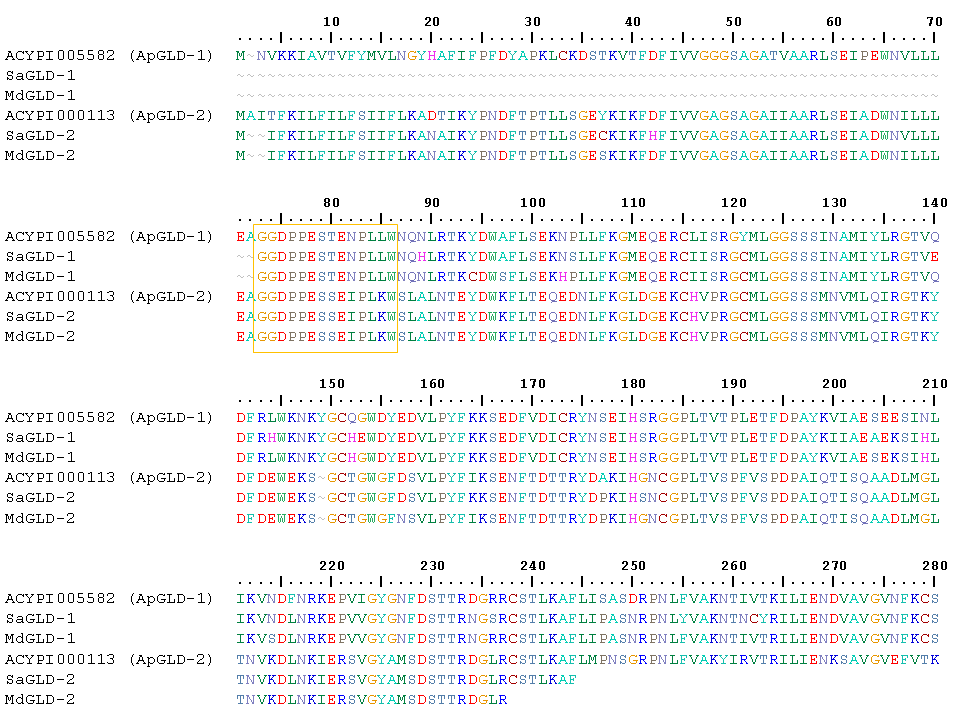

Supplement: Figure S1 — Predicted amino acid alignment for the two saliva associated GLD paralogues for A. pisum (ApGLD-1 and ApGLD-2), S. avenae (SaGLD-1 and SaGLD-2) and M. dirhodum (MdGLD-1 and MdGLD-2). The box indicates the antigenic peptide sequence that was chosen for antibody design. (DOCX) [file pone.0057413.s001.docx]

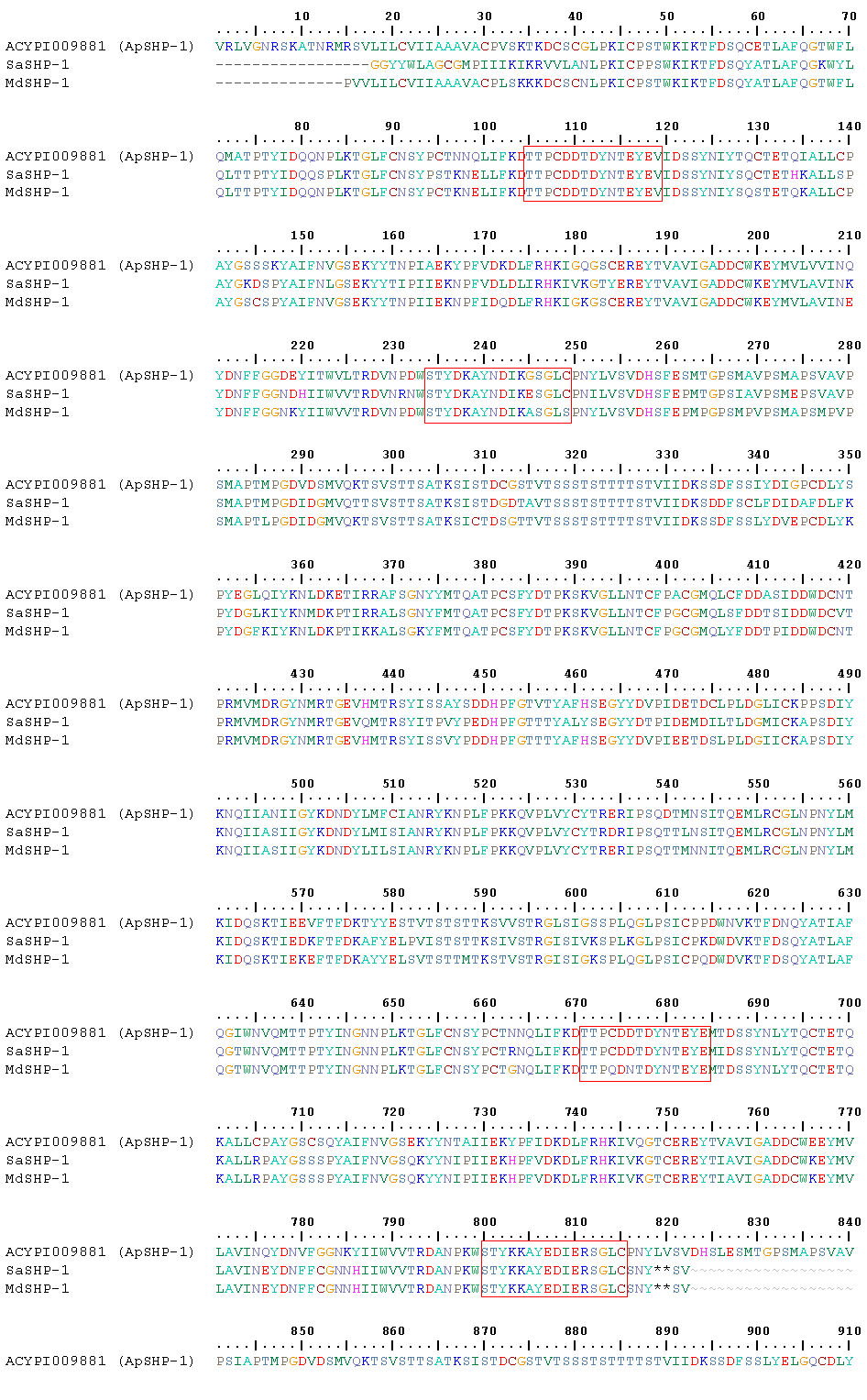
**Figure S2**

Supplement: Figure S2 — Predicted amino acid alignment for ACYPI009881 for A. pisum , S. avenae and M. dirhodum . The boxes indicating the antigenic peptide sequences chosen for antibody design are highlighted. (DOCX) [file pone.0057413.s002.docx]

**Figure S3.**

*
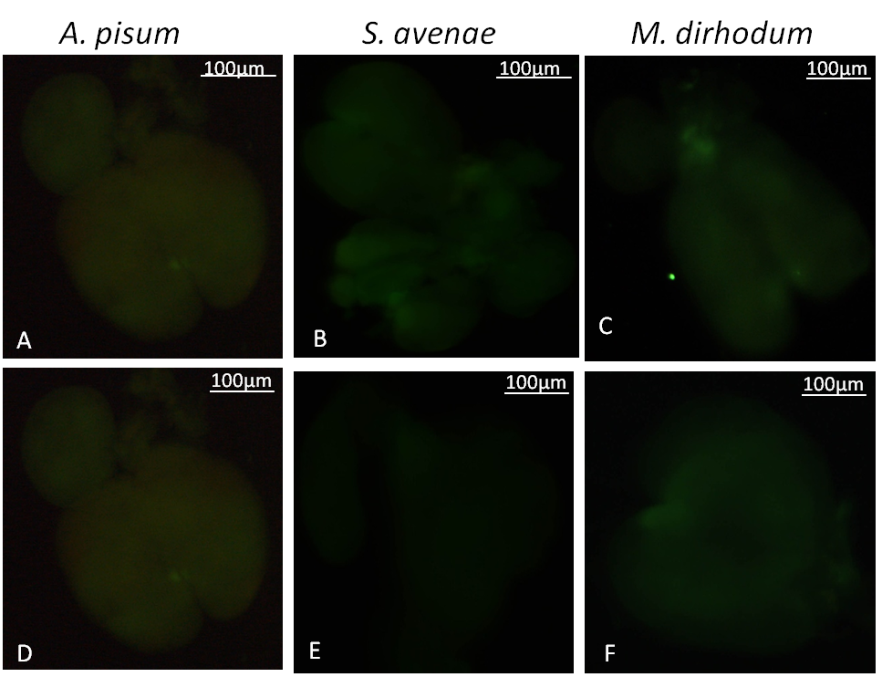
*

Supplement: Figure S3 — Localisation of SHP (A–C) and GLD (D–F) using secondary antibody as primary antibody on glands; (scale 100 µm for all pictures at 120×). (DOCX) [file pone.0057413.s003.docx]
