# Supplementary material for: Proteomic Profiling of Cereal Aphid Saliva Reveals Both Ubiquitous and Adaptive Secreted Proteins
Source: PLoS One. 2013 Feb 27;8(2):e57413. doi: 10.1371/journal.pone.0057413 (PMC3584018; doi:10.1371/journal.pone.0057413)
Supplement: Table S1 — Primer sequences and optimised annealing temperatures for M. dirhodum and S. avenae saliva associated GLDs and putative sheath protein. (DOCX) [file pone.0057413.s004.docx]

**Table S1.** Primer sequences and optimised annealing temperatures for *M. dirhodum* and *S. avenae* saliva associated GLDs and putative sheath protein.

| **Gene** | **Primer combination** | **Primers** | **Primer Sequences** | **Optimised Annealing Temperature (°C)** |
| --- | --- | --- | --- | --- |
| Putative Sheath Protein | ApSA-F1/R1 | ApSA-F1 | 5'-CAGTTCGTCTTGTTGGGAACC-3' | 65 |
|  |  | ApSA-R1 | 5'-CACACTTGGTGCCATTGAAGG-3' |  |
|  |  |  |  |  |
|  | ApSA-F2/R2 | ApSA-F2 | 5'-GGTAAGTGTTGATCATTCG-3' | 50 |
|  |  | ApSA-R2 | 5'-CCAATGCTGCTGAGTCCACGTG-3' |  |
|  |  |  |  |  |
|  | ApSA-F3/R3 | ApSA-F3 | 5'-CTCTAGTCTATTGTTATACCAG-3' | 55 |
|  |  | ApSA-R3 | 5'-CTTGGTGCCATTGAAGGTCC-3' |  |
|  |  |  |  |  |
| GLD-1 | Ap5582-F1/R1 | Ap5582-F1 | 5'-GAAGCTGGAGGTGATCCACC-3' | 60 |
|  |  | Ap5582-R1 | 5'-GAGAGCATAAGAAGCTGTGG-3' |  |
|  |  |  |  |  |
| GLD-2 | Ap113-F1/R1 | Ap113-F1 | 5'-GAGGCAGGTGGTGATCCTCC -3' | 60 |
|  |  | Ap113-R1 | 5'-GATATCATAAGTAATTGAGG-3' |  |
