# Supplementary figures and images for: Correction: Proteomic profiling of cereal aphid saliva reveals both ubiquitous and adaptive secreted proteins
Source: PLoS One. 2024 May 23;19(5):e0304429. doi: 10.1371/journal.pone.0304429 (PMC11115246; doi:10.1371/journal.pone.0304429)

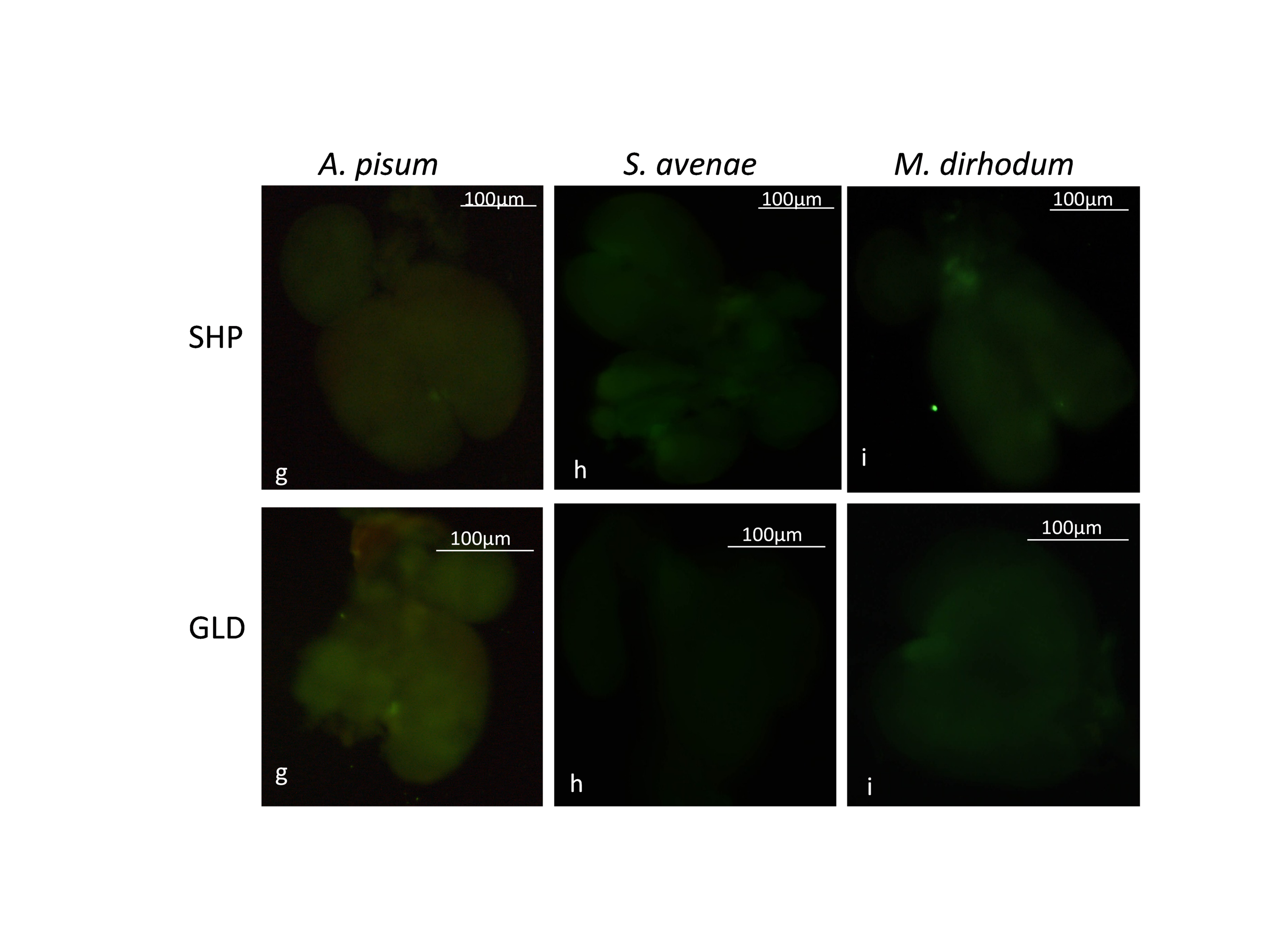

Supplement: S3 Fig — Localisation of SHP (A–C) and GLD (D–F) using secondary antibody as primary antibody on glands; (scale 100 µm for all pictures at 120×). (TIF) [file pone.0304429.s001.tif]

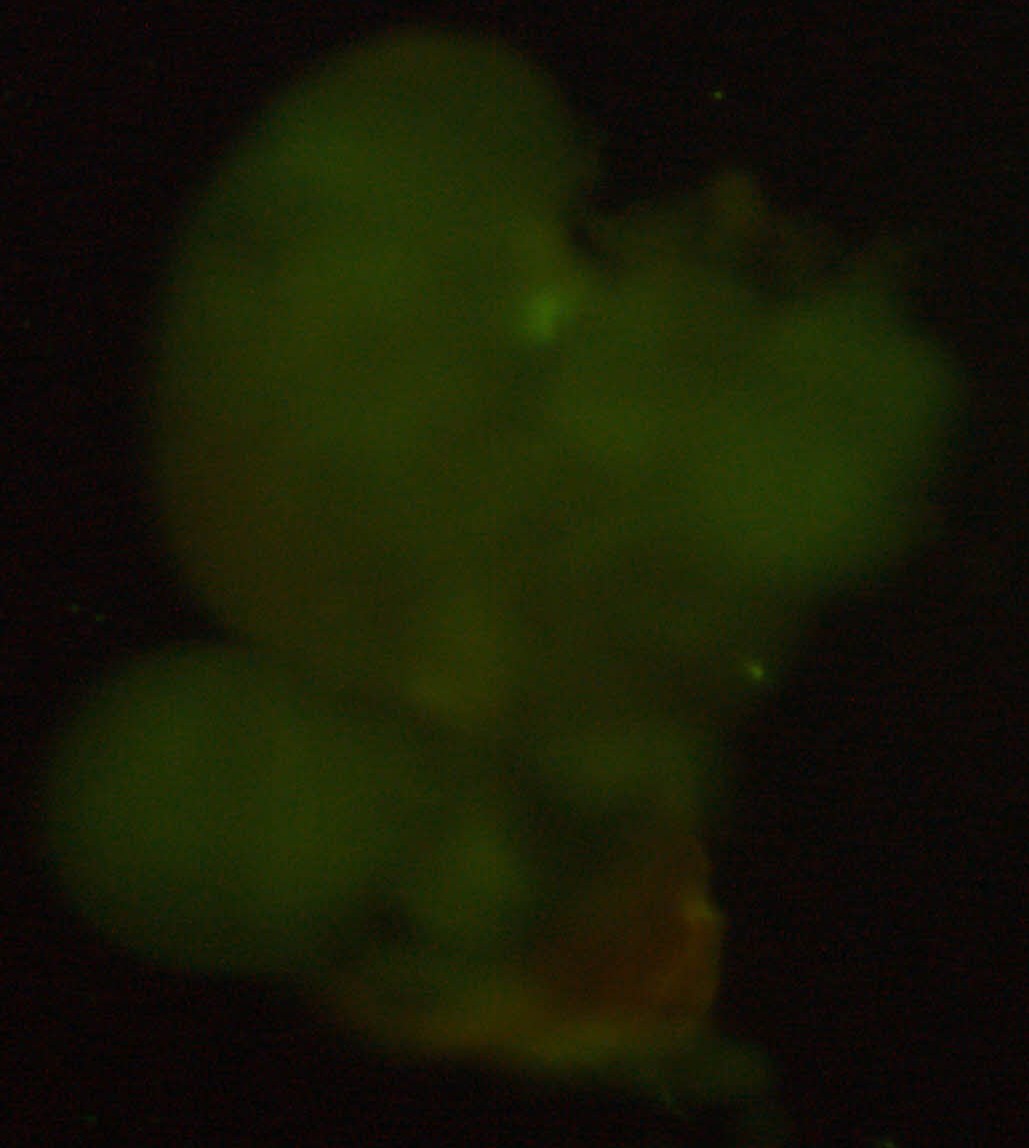

Supplement: S2 File — (ZIP) [file pone.0304429.s003.zip › File S2/a pisum 2ndry as primry r2.jpg]

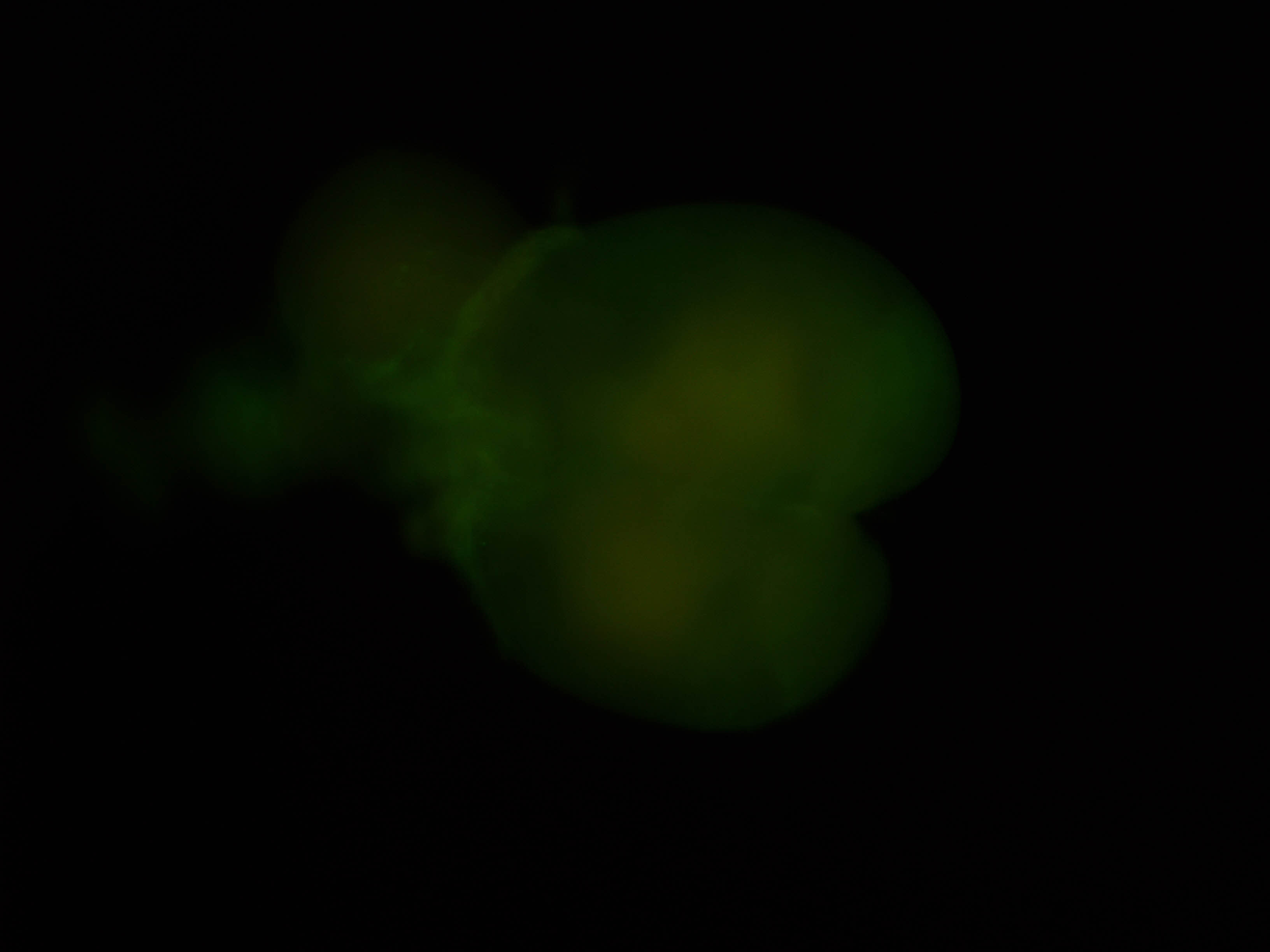

Supplement: S2 File — (ZIP) [file pone.0304429.s003.zip › File S2/ag23.jpg]

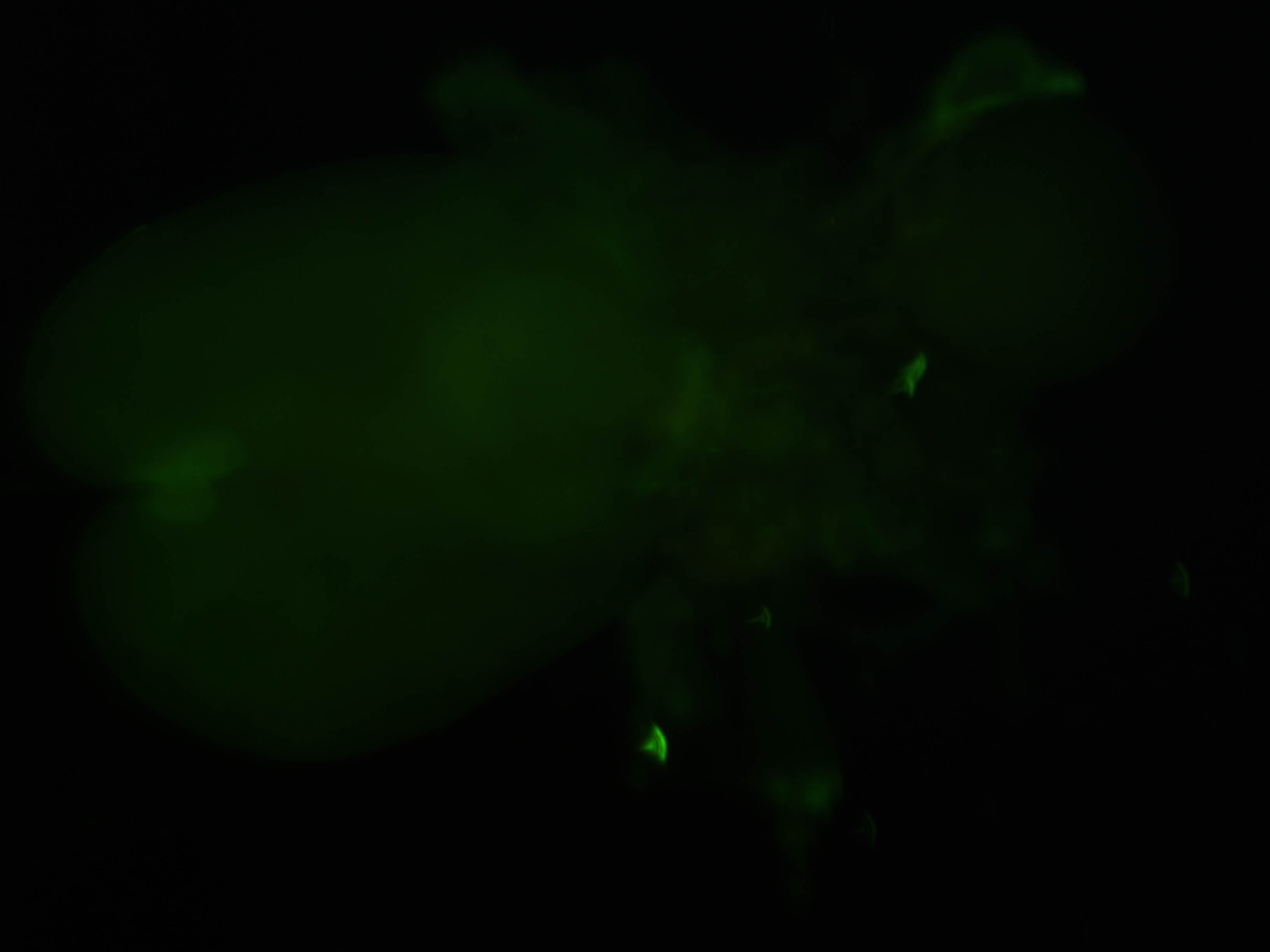

Supplement: S2 File — (ZIP) [file pone.0304429.s003.zip › File S2/ag6.jpg]

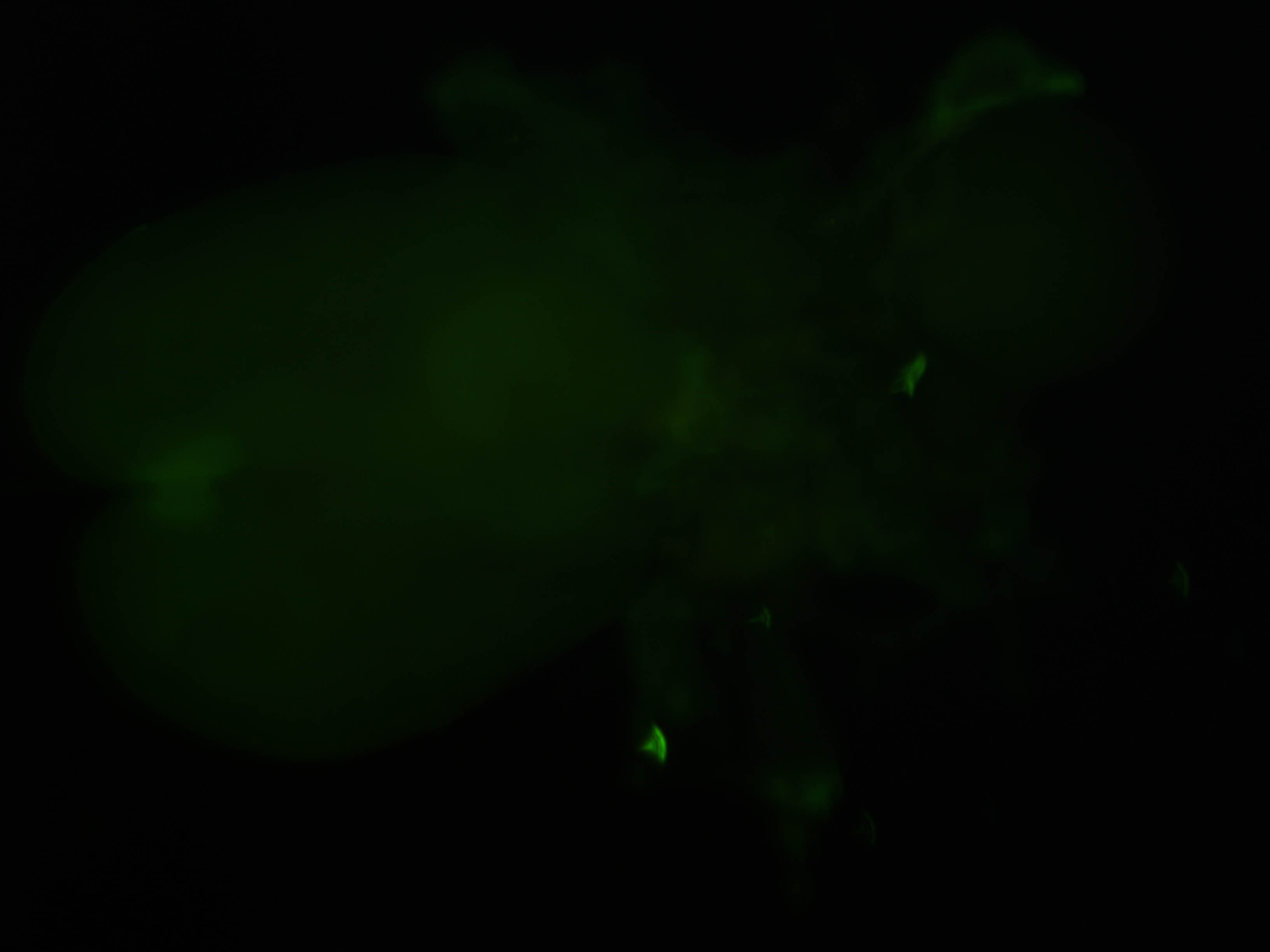

Supplement: S2 File — (ZIP) [file pone.0304429.s003.zip › File S2/ag7.jpg]

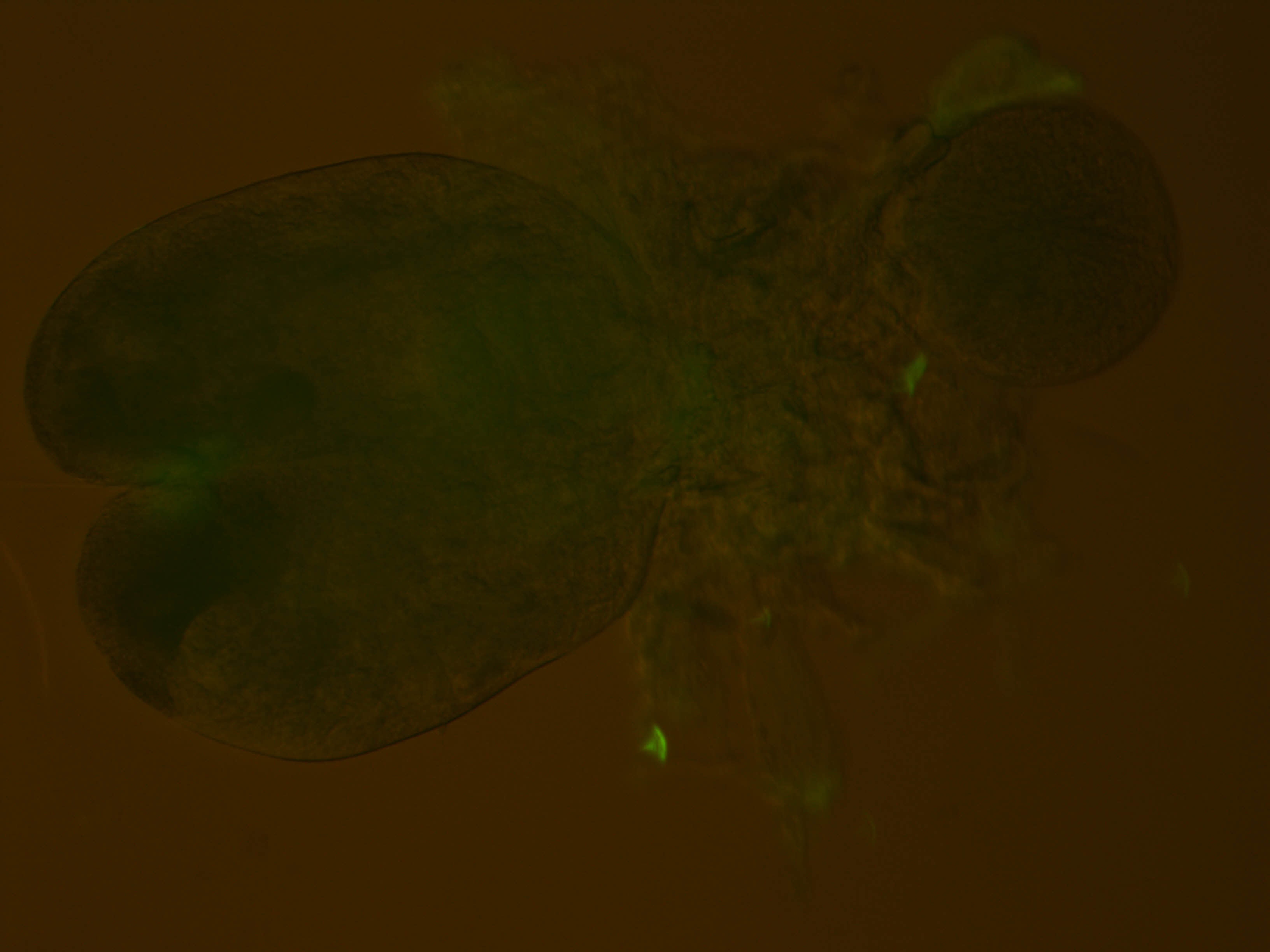

Supplement: S2 File — (ZIP) [file pone.0304429.s003.zip › File S2/ag8.jpg]

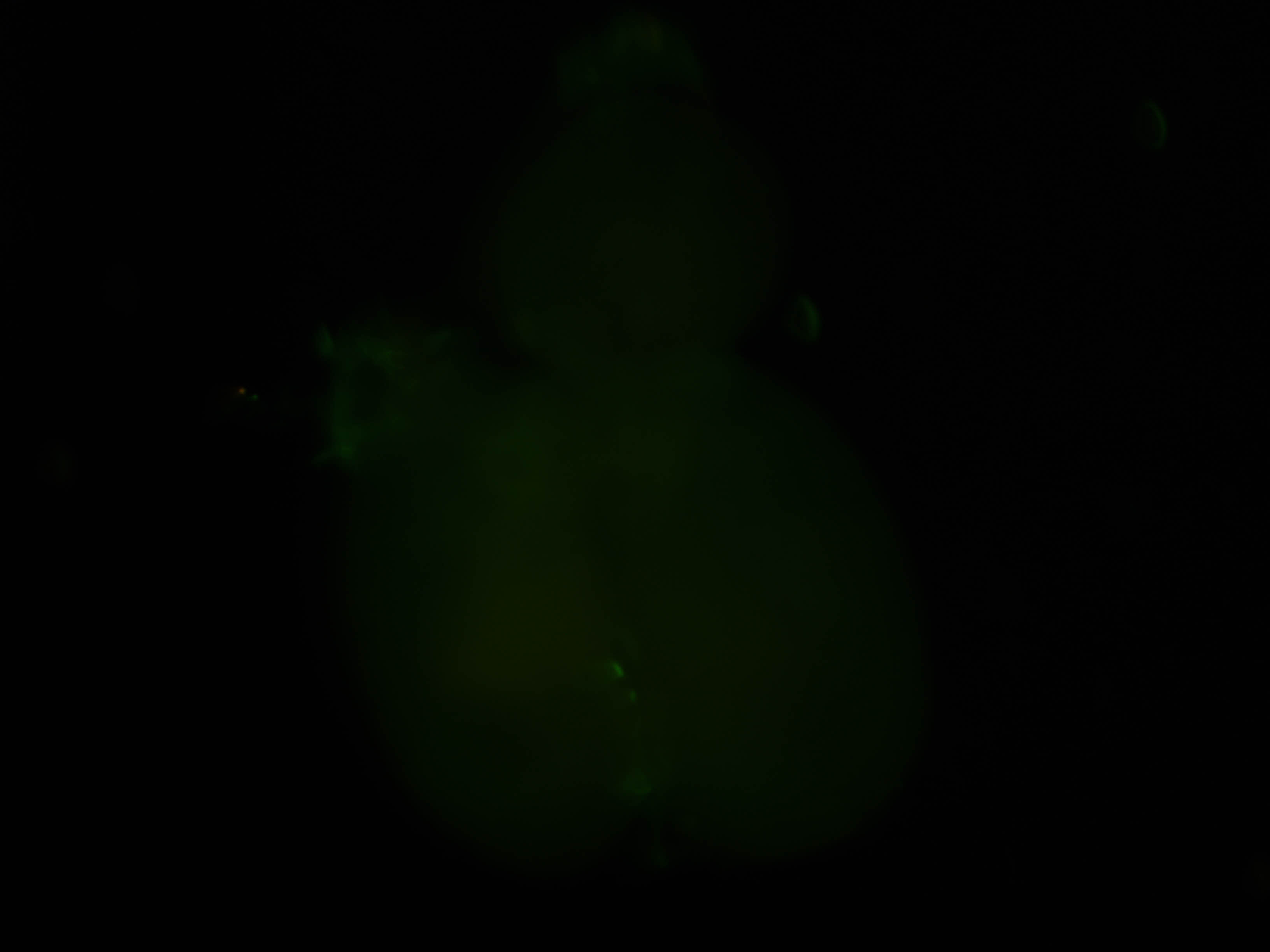

Supplement: S2 File — (ZIP) [file pone.0304429.s003.zip › File S2/bg.jpg]

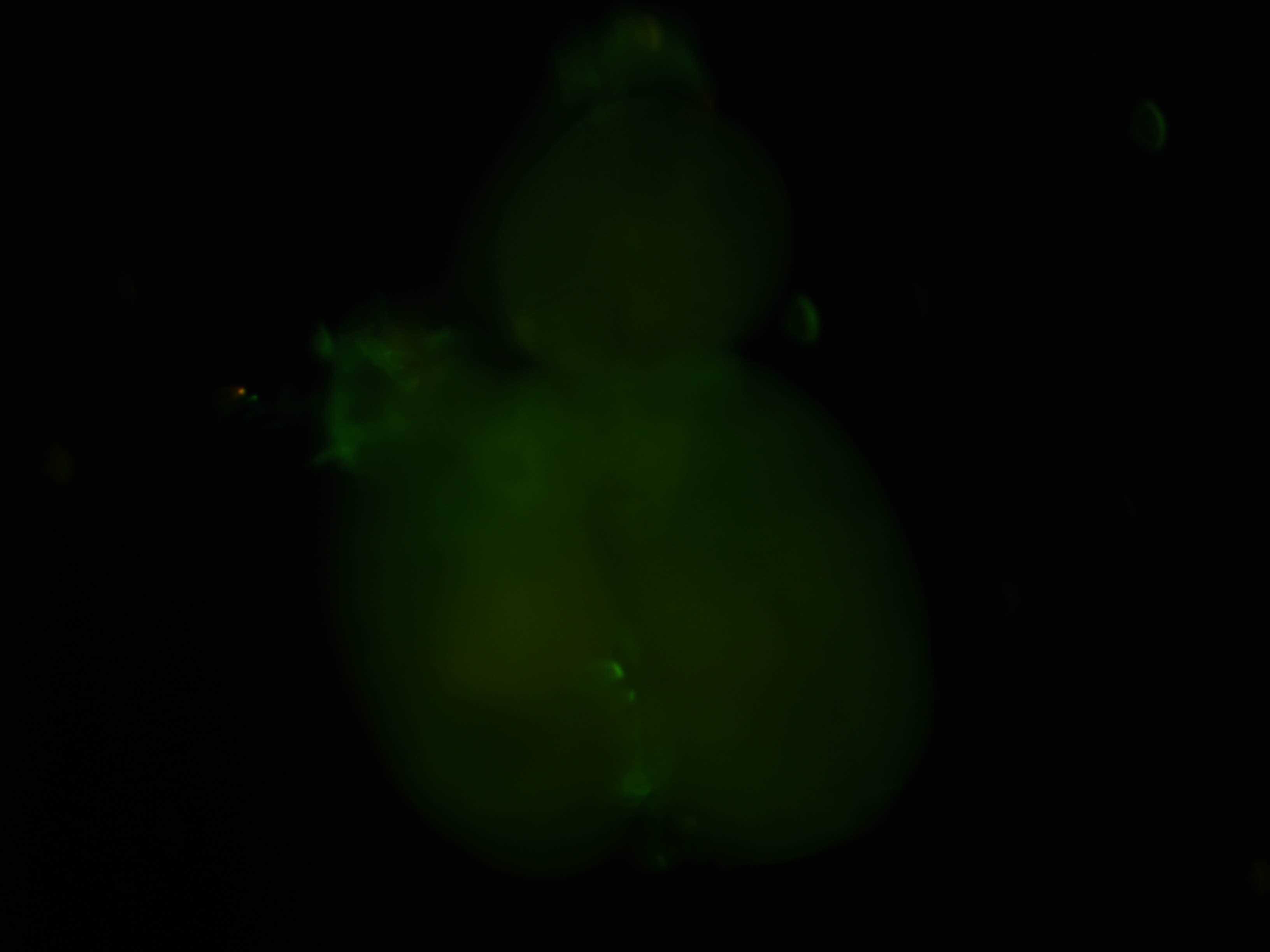

Supplement: S2 File — (ZIP) [file pone.0304429.s003.zip › File S2/bg1.jpg]

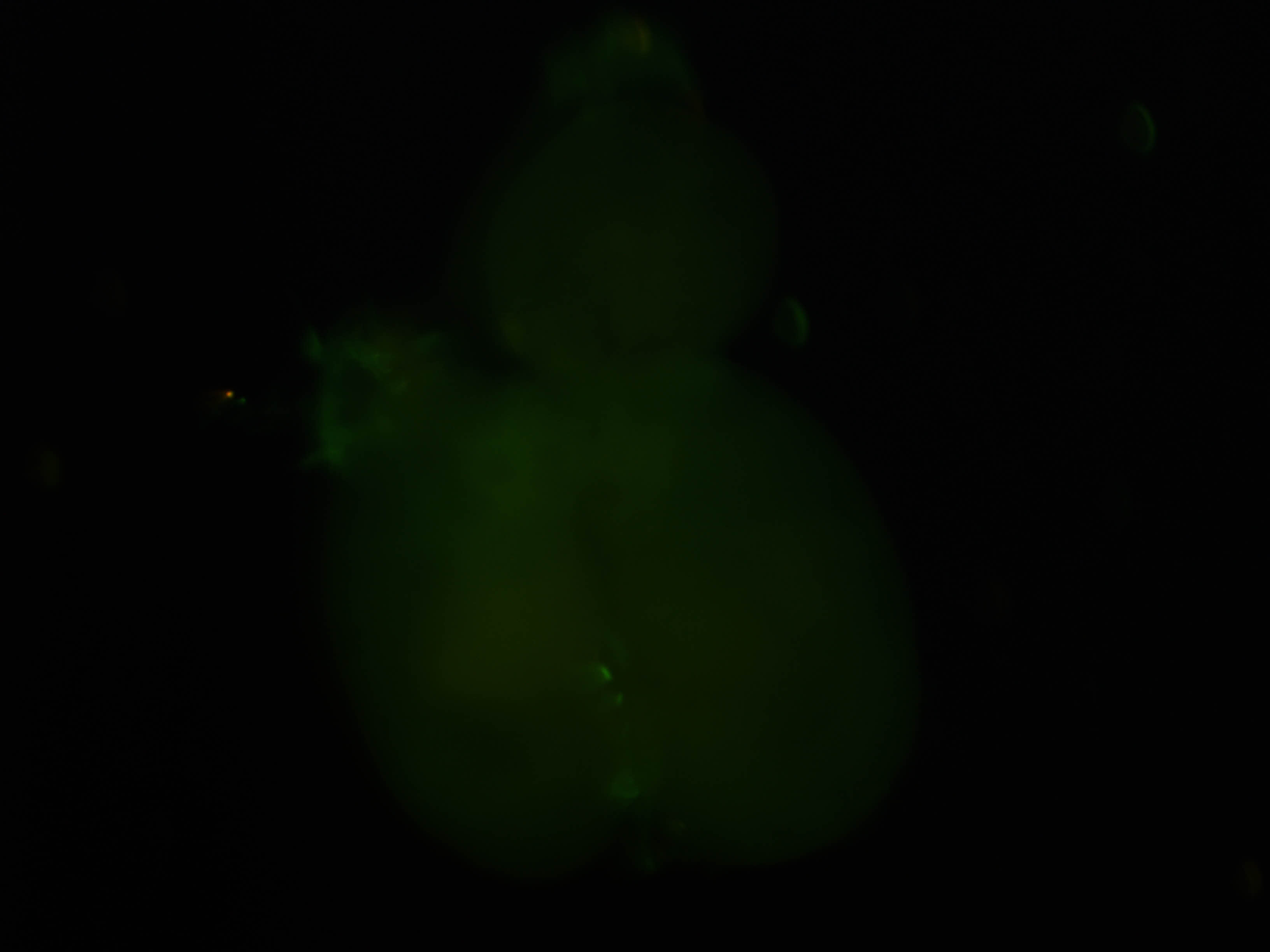

Supplement: S2 File — (ZIP) [file pone.0304429.s003.zip › File S2/bg2.jpg]

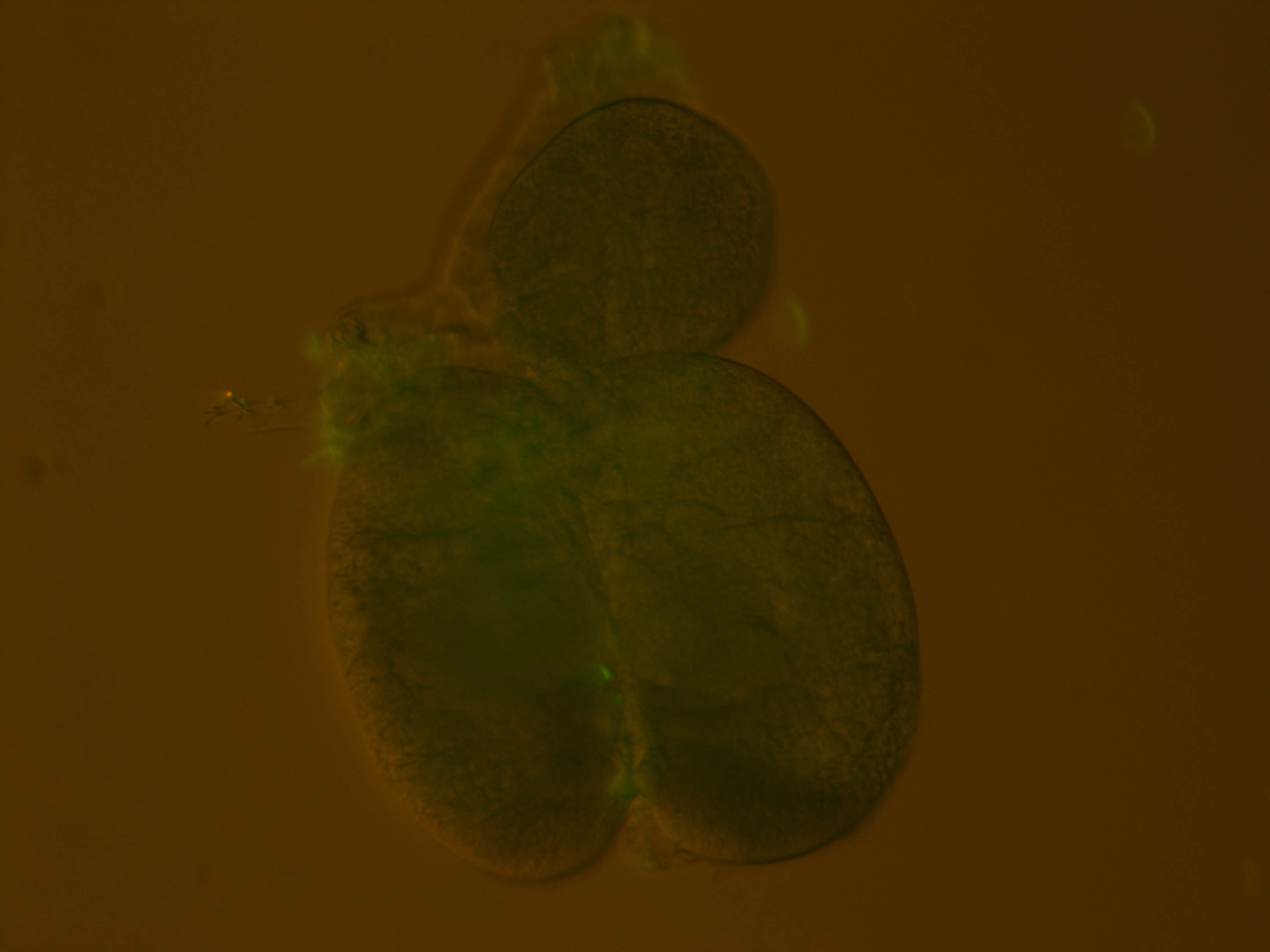

Supplement: S2 File — (ZIP) [file pone.0304429.s003.zip › File S2/bg3.jpg]

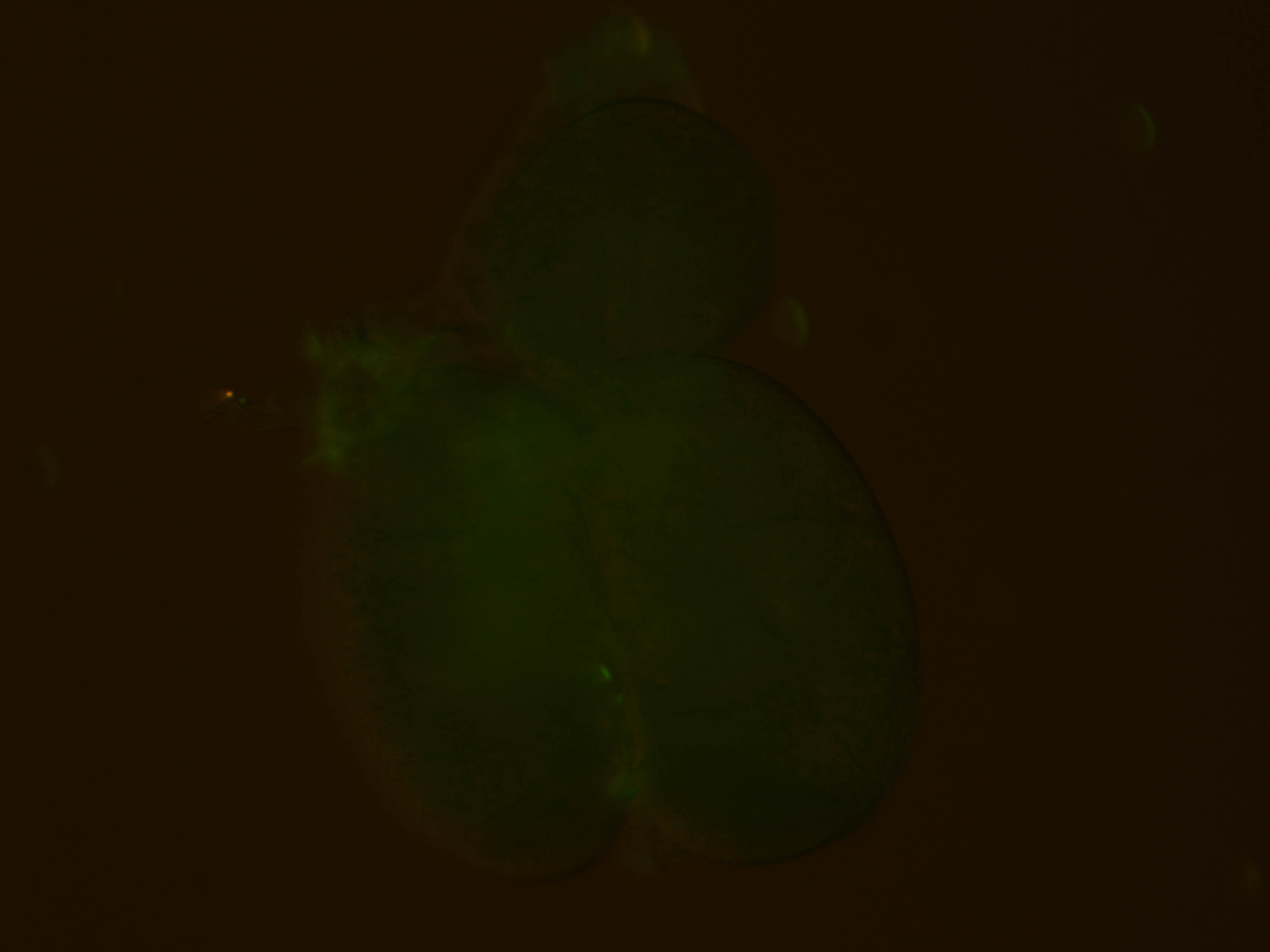

Supplement: S2 File — (ZIP) [file pone.0304429.s003.zip › File S2/bg4.jpg]

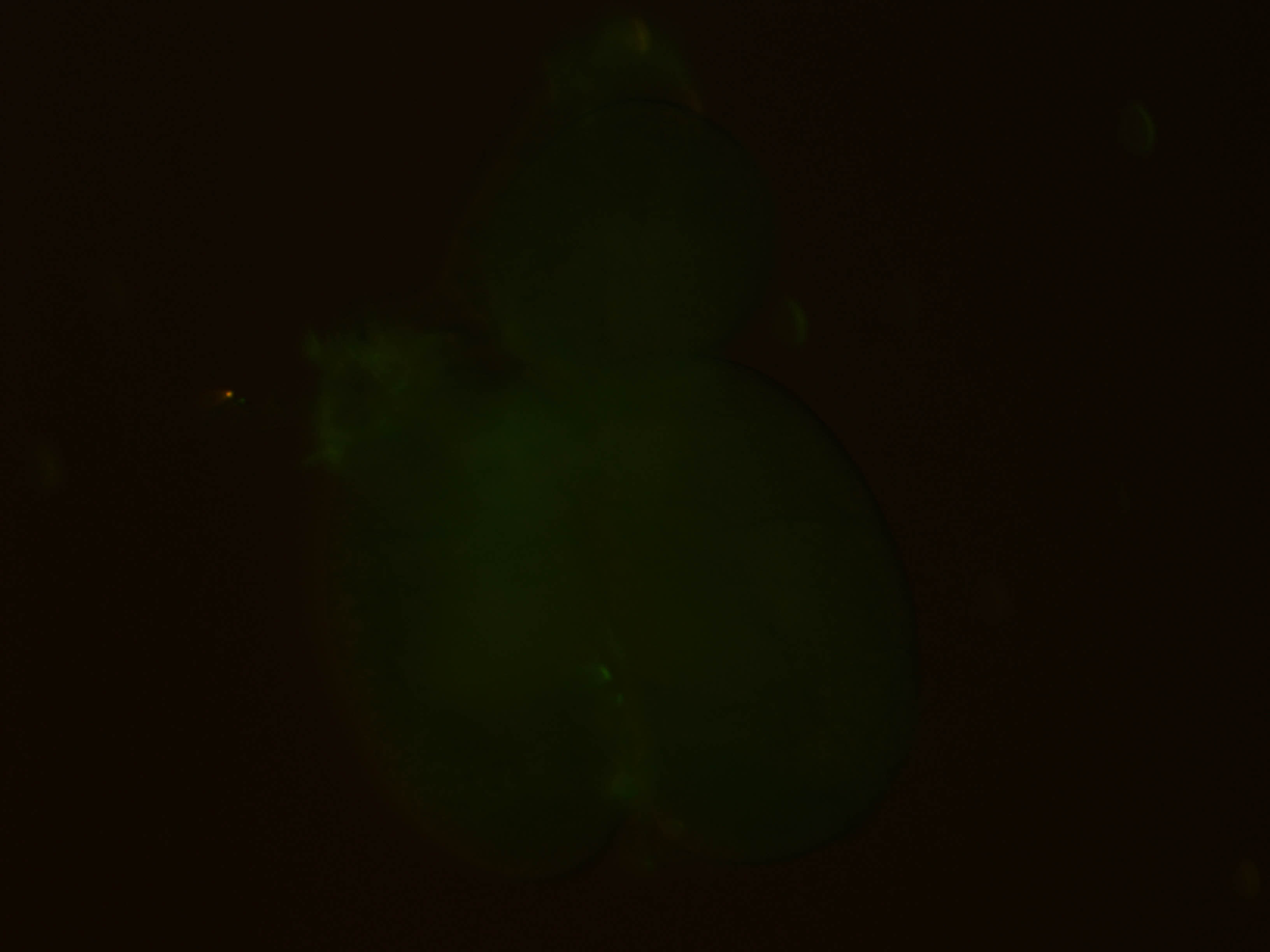

Supplement: S2 File — (ZIP) [file pone.0304429.s003.zip › File S2/bg5.jpg]

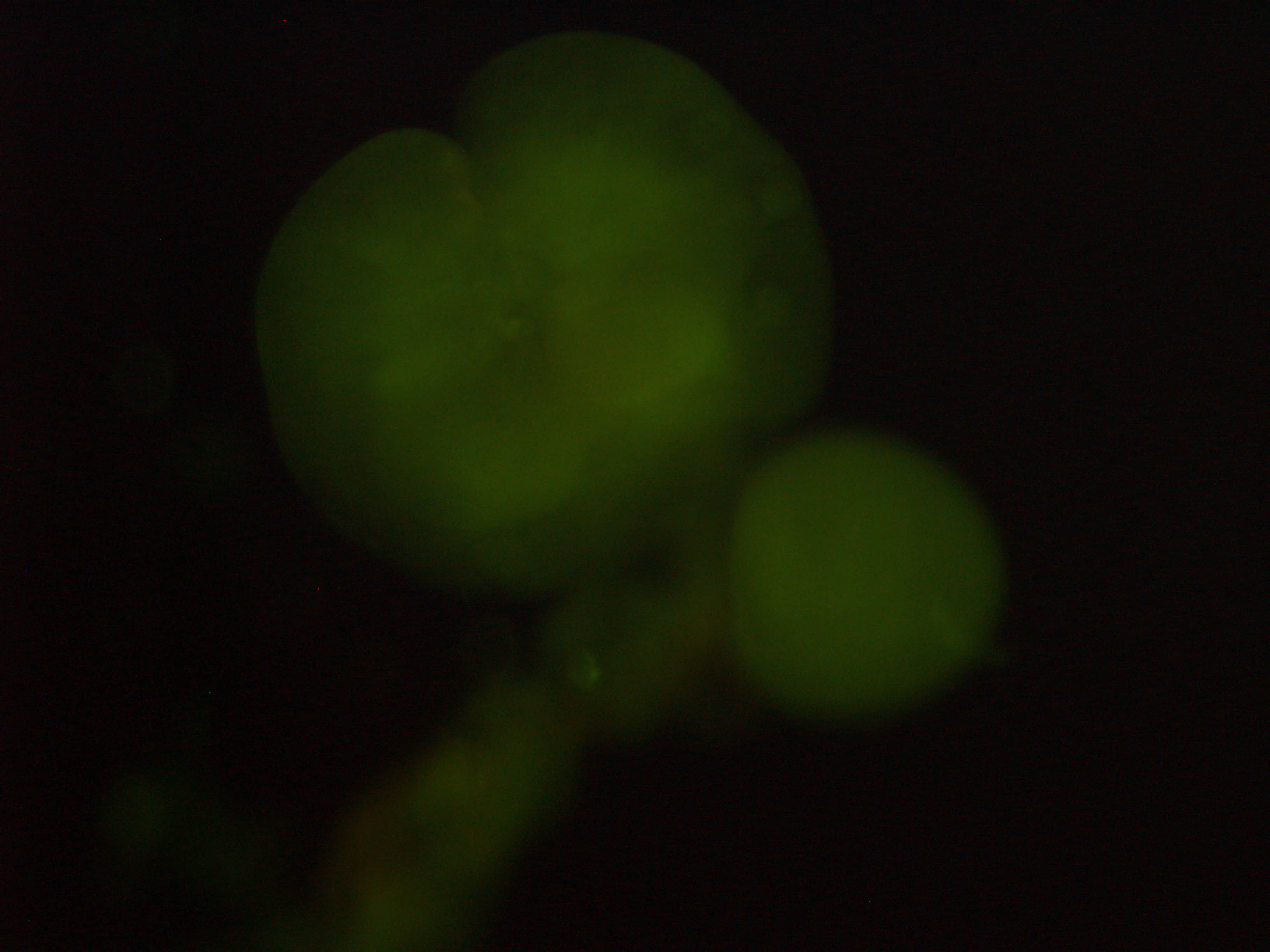

Supplement: S2 File — (ZIP) [file pone.0304429.s003.zip › File S2/c.jpg]

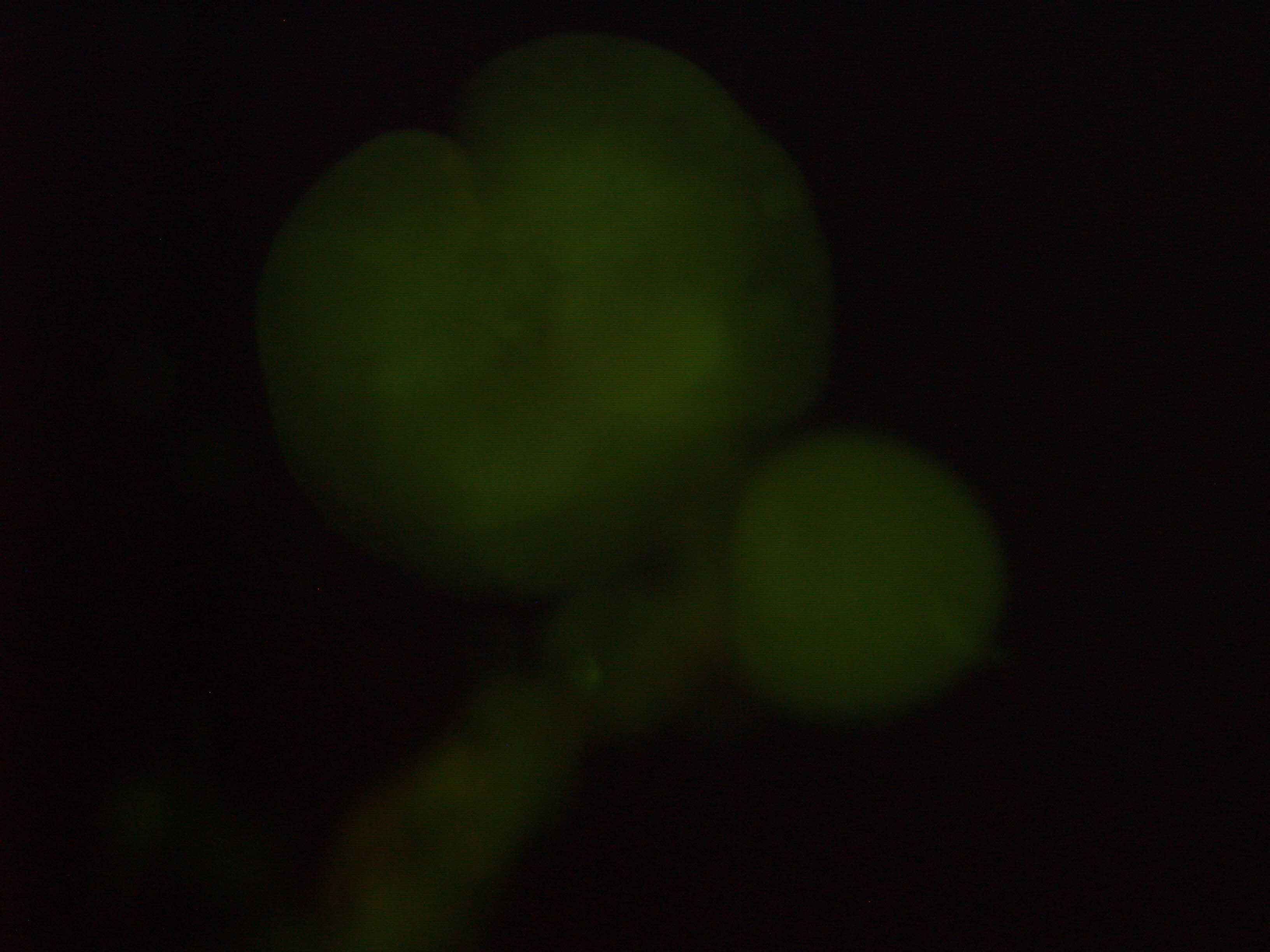

Supplement: S2 File — (ZIP) [file pone.0304429.s003.zip › File S2/c1.jpg]

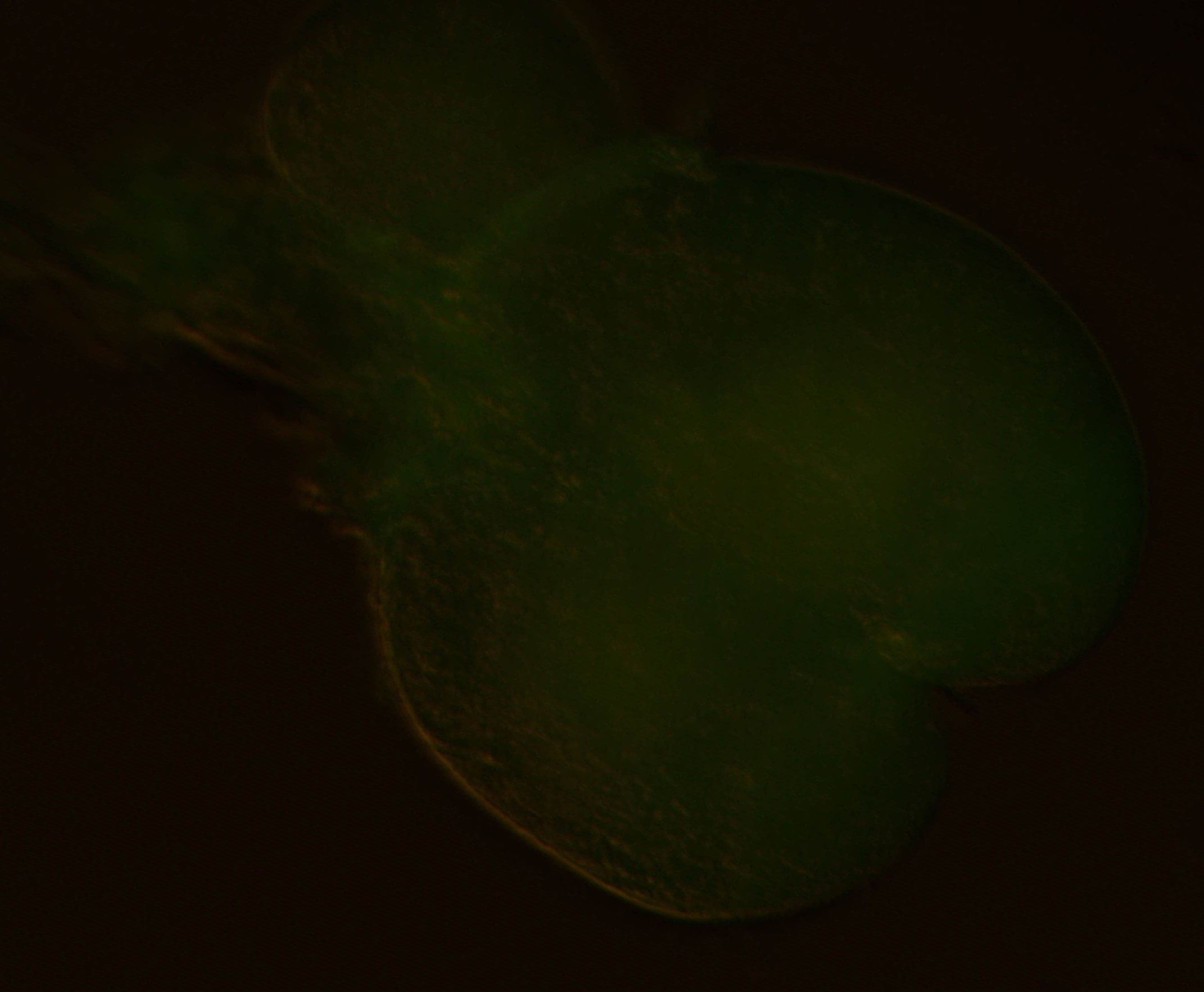

Supplement: S2 File — (ZIP) [file pone.0304429.s003.zip › File S2/cg25 - Copy.jpg]

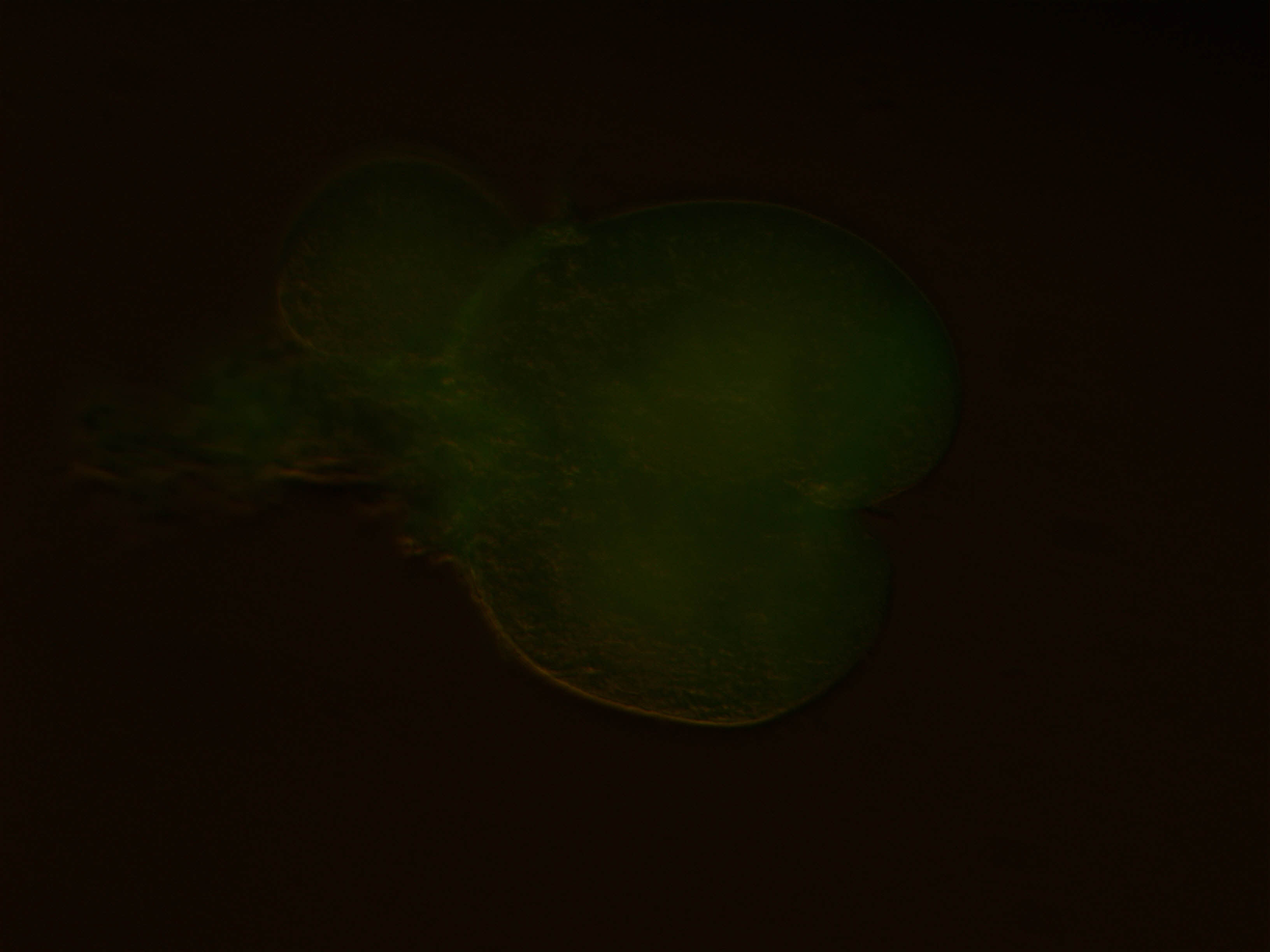

Supplement: S2 File — (ZIP) [file pone.0304429.s003.zip › File S2/cg25.jpg]

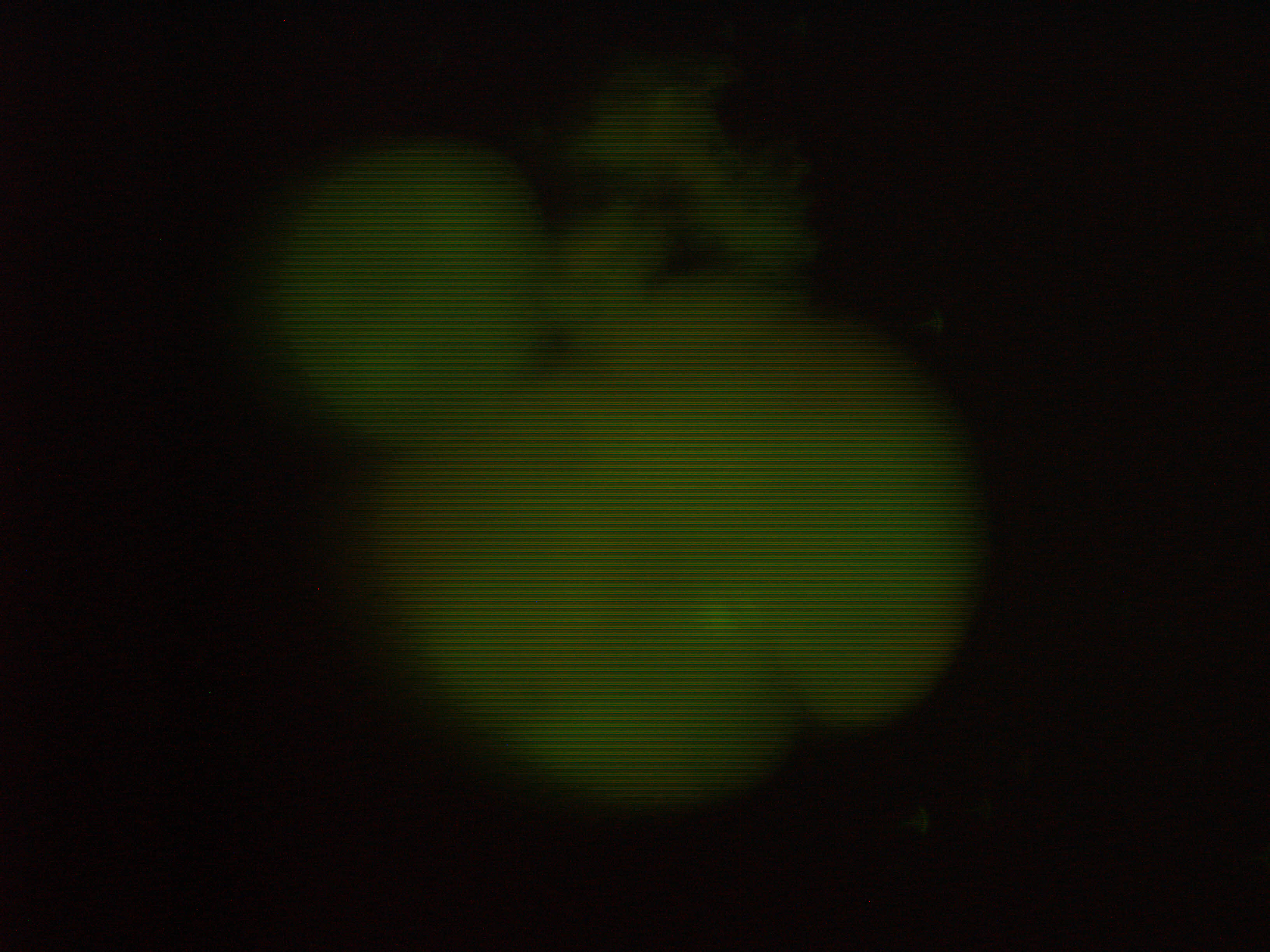

Supplement: S2 File — (ZIP) [file pone.0304429.s003.zip › File S2/g.jpg]

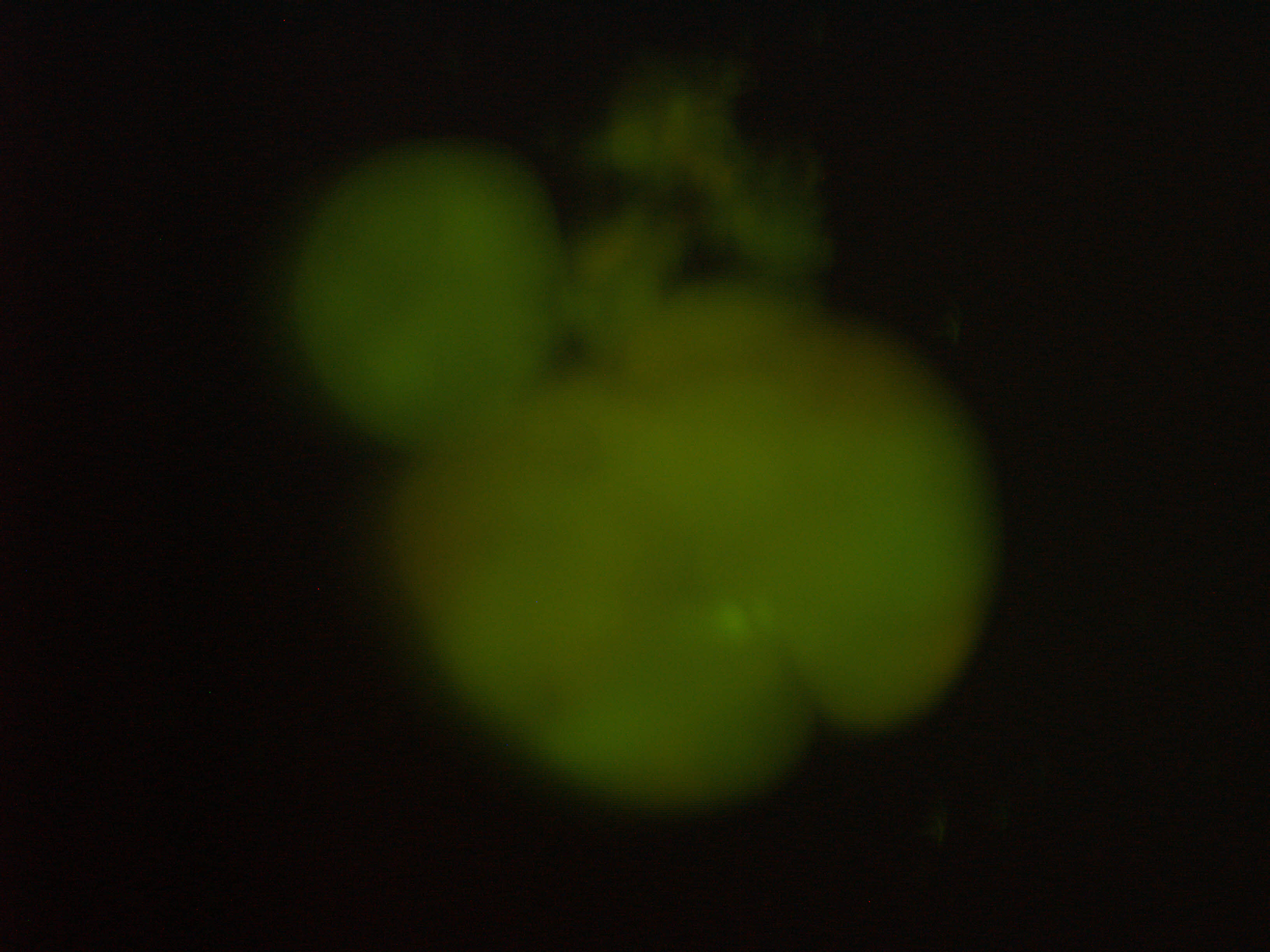

Supplement: S2 File — (ZIP) [file pone.0304429.s003.zip › File S2/g1.jpg]

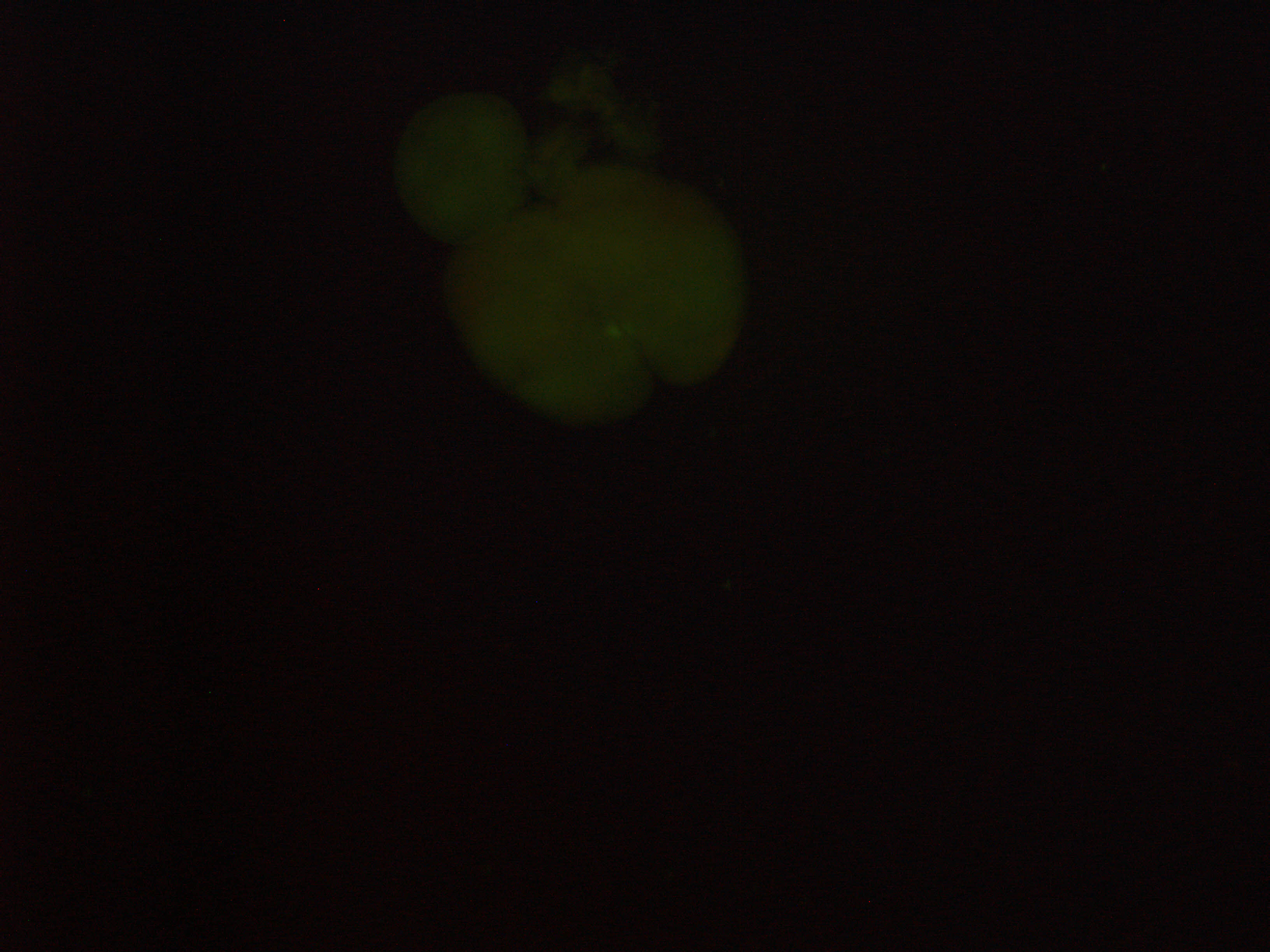

Supplement: S2 File — (ZIP) [file pone.0304429.s003.zip › File S2/g2.jpg]

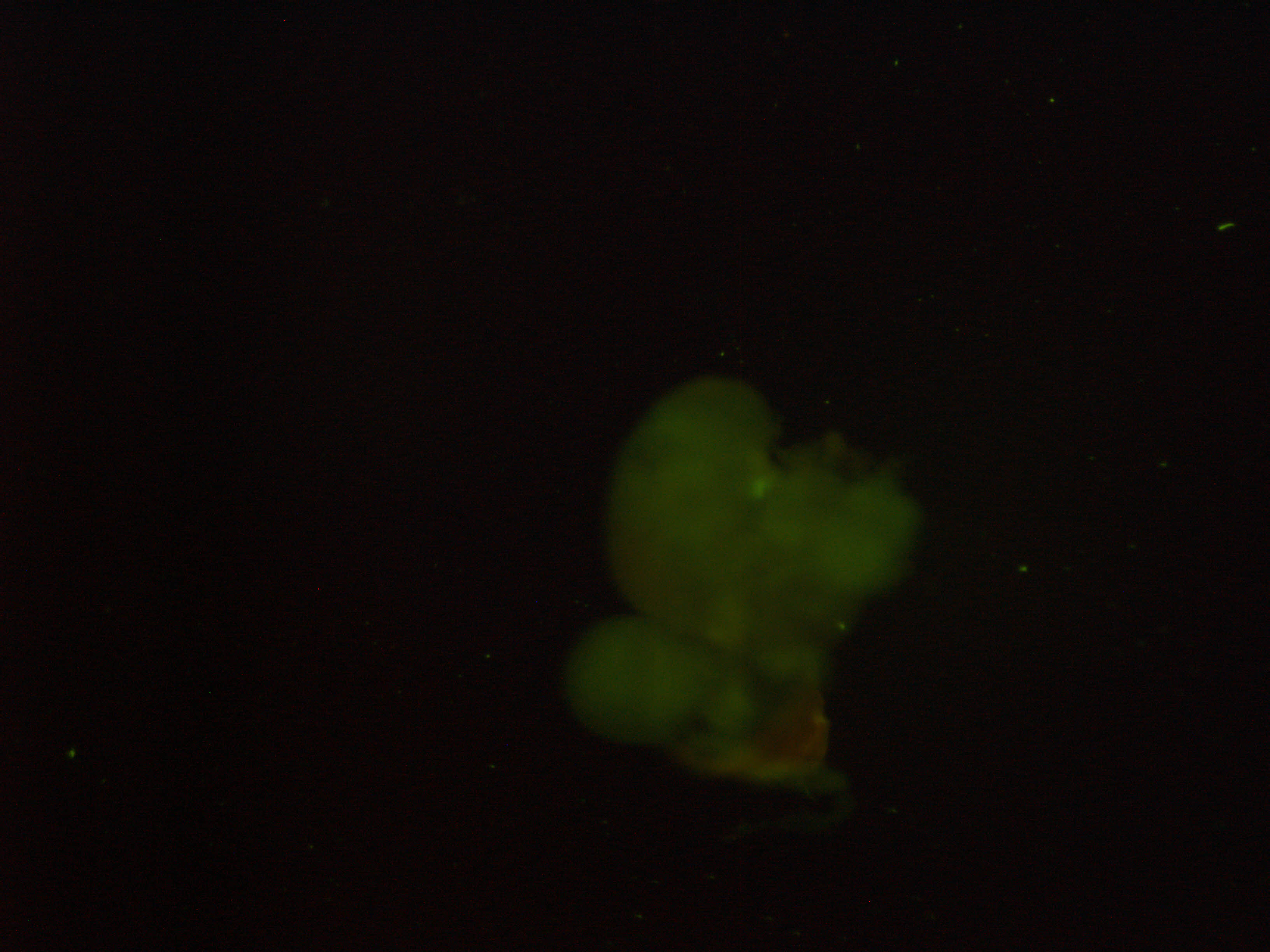

Supplement: S2 File — (ZIP) [file pone.0304429.s003.zip › File S2/g4.jpg]
