# Supplementary material for: Correction: Proteomic profiling of cereal aphid saliva reveals both ubiquitous and adaptive secreted proteins
Source: PLoS One. 2024 May 23;19(5):e0304429. doi: 10.1371/journal.pone.0304429 (PMC11115246; doi:10.1371/journal.pone.0304429)
Supplement: S1 File — (PPTX) [file pone.0304429.s002.pptx]

## Slide 1
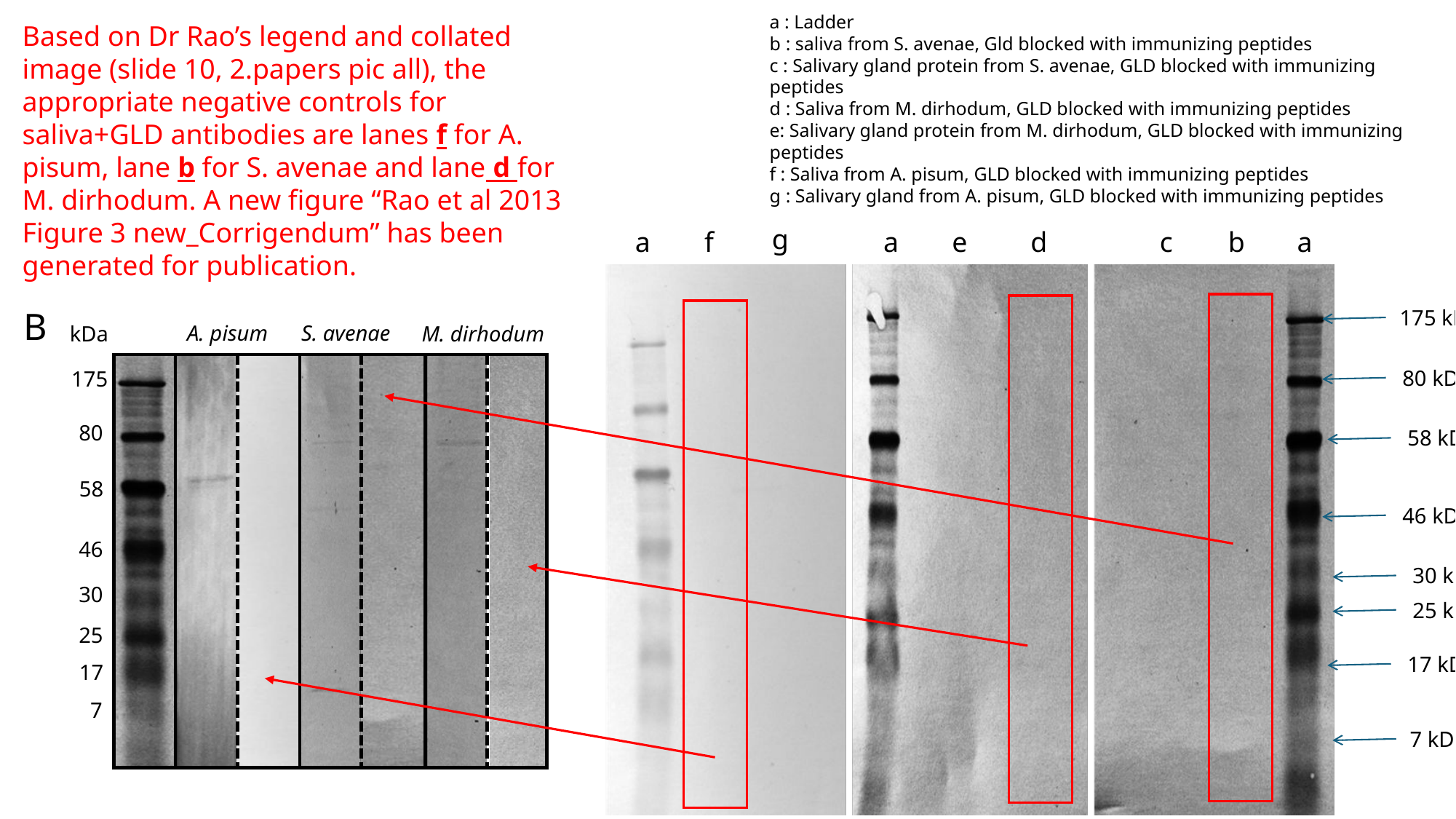

a : Ladder
b : saliva from S. avenae, Gld blocked with immunizing peptides
c : Salivary gland protein from S. avenae, GLD blocked with immunizing peptides
d : Saliva from M. dirhodum, GLD blocked with immunizing peptides
e: Salivary gland protein from M. dirhodum, GLD blocked with immunizing peptides
f : Saliva from A. pisum, GLD blocked with immunizing peptides
g : Salivary gland from A. pisum, GLD blocked with immunizing peptides
Based on Dr Rao’s legend and collated image (slide 10, 2.papers pic all), the appropriate negative controls for saliva+GLD antibodies are lanes f for A. pisum, lane b for S. avenae and lane d for M. dirhodum. A new figure “Rao et al 2013 Figure 3 new_Corrigendum” has been generated for publication.
g
a
f
a
e
d
c
b
a
175 kD
80 kD
58 kD
46 kD
30 kD
25 kD
17 kD
7 kD
B
A. pisum
S. avenae
M. dirhodum
kDa
175
80
58
46
30
25
17
7

## Slide 2
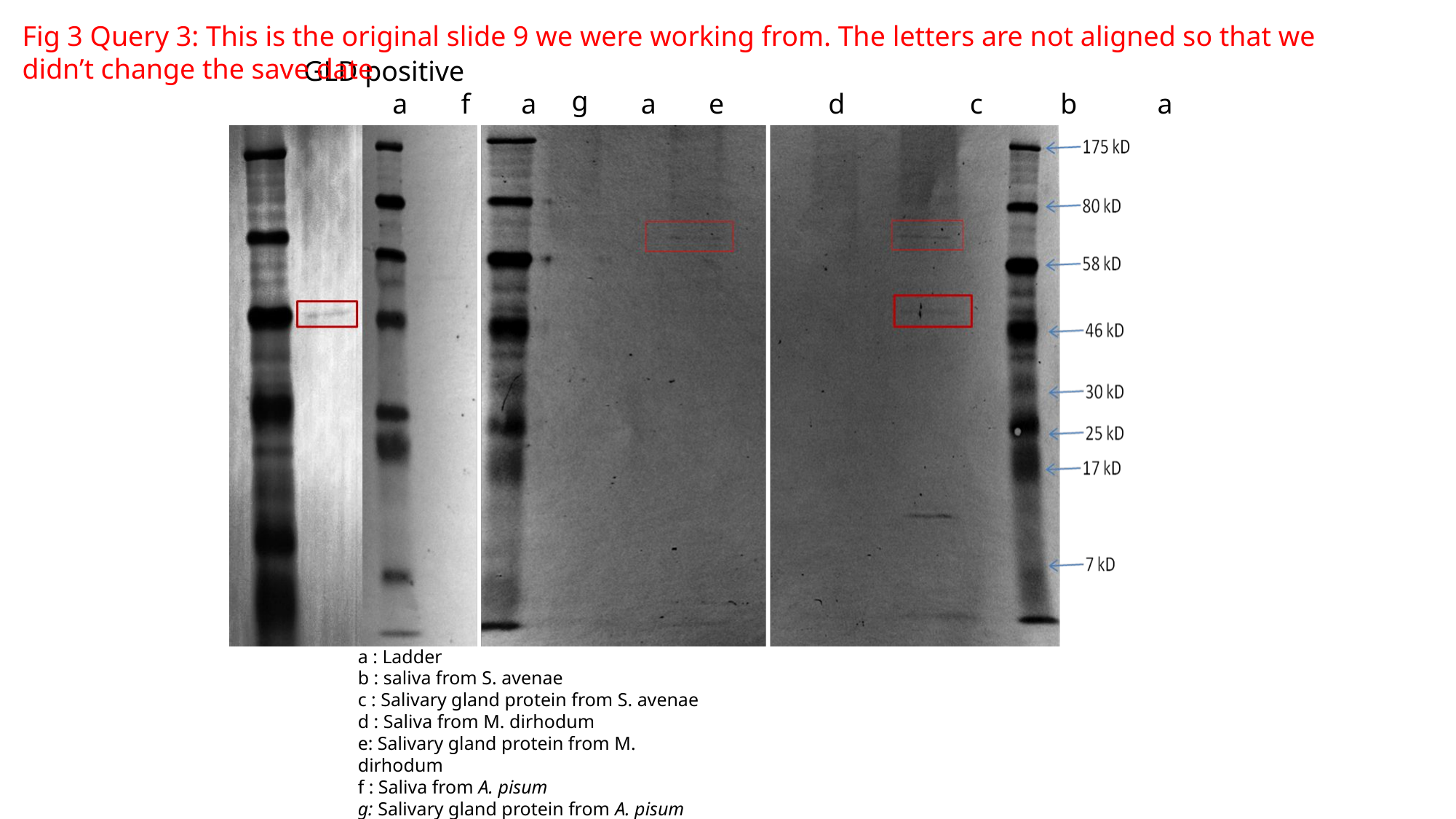

Fig 3 Query 3: This is the original slide 9 we were working from. The letters are not aligned so that we didn’t change the save date
GLD positive
g
a
f
a
a
e
d
c
b
a
a : Ladder
b : saliva from S. avenae
c : Salivary gland protein from S. avenae
d : Saliva from M. dirhodum
e: Salivary gland protein from M. dirhodum
f : Saliva from A. pisum
g: Salivary gland protein from A. pisum

## Slide 3
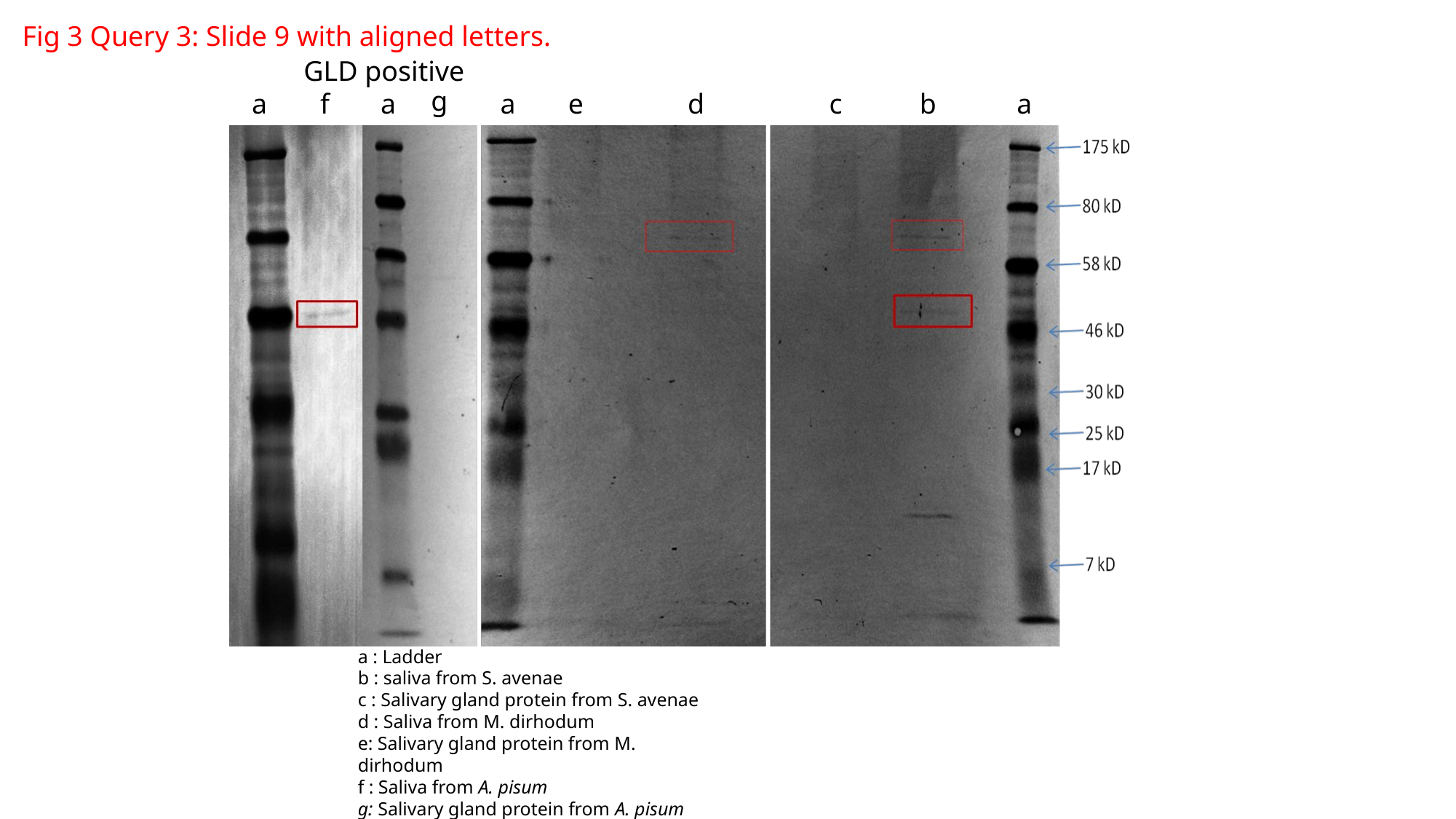

Fig 3 Query 3: Slide 9 with aligned letters.
GLD positive
g
a
f
a
a
e
d
c
b
a
a : Ladder
b : saliva from S. avenae
c : Salivary gland protein from S. avenae
d : Saliva from M. dirhodum
e: Salivary gland protein from M. dirhodum
f : Saliva from A. pisum
g: Salivary gland protein from A. pisum

## Slide 4
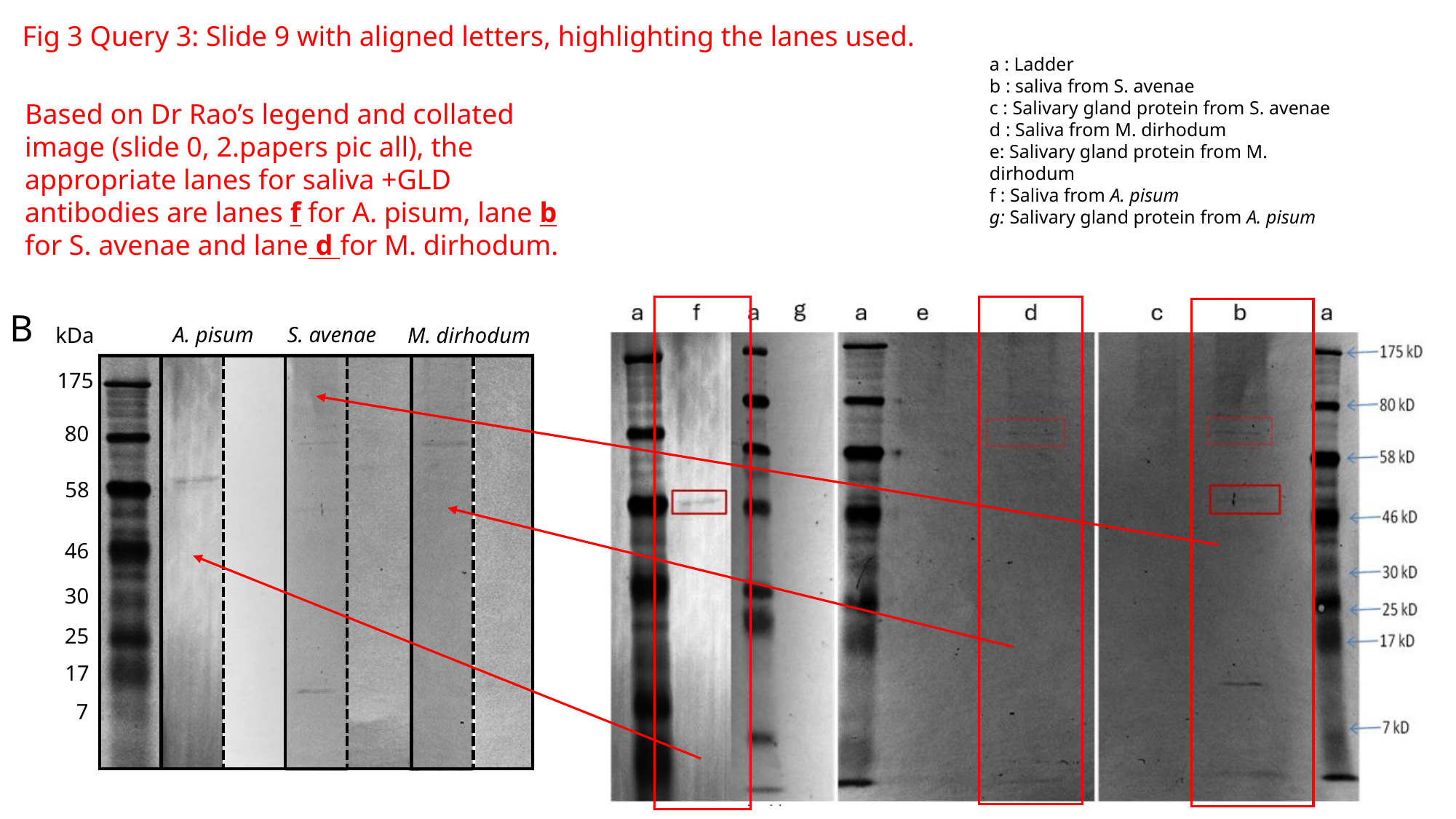

Fig 3 Query 3: Slide 9 with aligned letters, highlighting the lanes used.
a : Ladder
b : saliva from S. avenae
c : Salivary gland protein from S. avenae
d : Saliva from M. dirhodum
e: Salivary gland protein from M. dirhodum
f : Saliva from A. pisum
g: Salivary gland protein from A. pisum
Based on Dr Rao’s legend and collated image (slide 0, 2.papers pic all), the appropriate lanes for saliva +GLD antibodies are lanes f for A. pisum, lane b for S. avenae and lane d for M. dirhodum.
B
A. pisum
S. avenae
M. dirhodum
kDa
175
80
58
46
30
25
17
7
